# Life Cycle Assessment and Critical Raw Materials Analysis of Innovative Palladium-Substituted Membranes for Hydrogen Separation

**DOI:** 10.3390/membranes15100310

**Published:** 2025-10-13

**Authors:** Ali Mohtashamifar, Simone Battiston, Stefano Fasolin, Stefania Fiameni, Francesca Visentin, Simona Barison

**Affiliations:** Institute of Condensed Matter Chemistry and Technologies for Energy (ICMATE), National Research Council of Italy (CNR), C.So Stati Uniti 4, 35127 Padua, Italy; alimohtashamifar@cnr.it (A.M.); stefano.fasolin@cnr.it (S.F.); stefania.fiameni@cnr.it (S.F.); francesca.visentin@cnr.it (F.V.); simona.barison@cnr.it (S.B.)

**Keywords:** life cycle assessment, uncertainty analysis, critical raw materials, hydrogen separation, membranes

## Abstract

Palladium-based membranes for hydrogen separation offer the most promising gas permeation and selectivity, but their large-scale application has been limited due to the high environmental burdens and criticality of palladium. Herein, the possibility of substituting Pd with candidate elements in the composition of metallic micro-scale membranes (with permeability in the range of 5–50 × 10^−12^ mol m^–1^ Pa^–1^ s^−1^) deposited via High Power Impulse Magnetron Sputtering was investigated. This study proposed an innovative framework for a more comprehensive investigation of the sustainability challenges related to this lab-scale technology by integrating Life Cycle Assessment (LCA) and criticality analyses, thereby supporting materials selection efforts. First, the criticality status of several elements used in hydrogen separation membranes was screened with two different approaches. Furthermore, the environmental impacts of novel membrane compositions were compared with a high Pd-content reference membrane (Pd_77_Ag_23_) through cradle-to-gate LCA. For robust LCA modeling, uncertainty analysis was performed via Monte Carlo simulation, exploiting errors estimated for both primary and secondary data. A direct relationship was identified between the Pd content in membranes and the associated environmental impacts. VPd proved to be a promising candidate by exhibiting lower total impacts than the PdAg (65% or 71% considering thickness of 3.16 µm or permeance of 2.03 × 10^−6^ mol m^−2^ Pa^−1^ s^−1^, respectively).

## 1. Introduction

Fossil fuels currently supply over 80% of global energy consumption, and their combustion accounts for two-thirds of the greenhouse gas emissions [1]. The increasing depletion of these non-renewable resources prompts an urgent need to explore more sustainable alternatives [2]. In this context, hydrogen has been an attractive energy vector offering a pathway to decarbonize industrial processes [3,4].

Hydrogen production involves steam reforming from fossil or biomass fuels, followed by further purification to remove undesired by-products (CO, CO_2_, H_2_S, and NH_3_) [5]. Pure hydrogen is essential for efficient fuel cell-based energy systems, highlighting the importance of developing separation technologies [2]. Over the past decades, H_2_ separation membranes have gained growing interest owing to their various advantages [6]. These membranes consist of materials with high hydrogen affinity to facilitate hydrogen transport across the matrix. The process is activated by pressure difference at sides of membrane and includes adsorption, dissociation, diffusion, and recombination of hydrogen molecules [7].

Transition metals such as palladium, vanadium, zirconium, and titanium are usually used in hydrogen-selective membranes to enhance permeability or prevent embrittlement [8]. Pd-based alloys are the preferred membranes (e.g., PdAg), as they demonstrate excellent permeation and selectivity due to palladium catalytic characteristics [2]. However, their large-scale application is hindered by high fabrication costs, sensitivity to embrittlement and surface poisoning, high environmental impacts, and raw material criticality [9]. Research is being conducted to replace Pd, even partially, to make hydrogen separation technology more sustainable, even at the expense of functional efficiency [10].

Raw material criticality can be assessed through several methods [11]. For instance, the European Commission has suggested guidelines to improve the sustainability of Critical Raw Materials (CRMs) supply chain [12]. According to the European Commission, CRMs are those that bear high economic importance and supply risk [11].

The European Commission has also proposed an assessment framework for Safe and Sustainable by Design (SSbD) materials [13], aimed at improving the sustainability of innovative processes. In this regard, Life Cycle Assessment (LCA) is an internationally standardized methodology [14], considered as the core element of environmental sustainability assessments [15]. It quantifies the potential impacts of products and processes on the environment and human health, considering the consumption of resources. Even though applying LCA to emerging technologies at low Technology Readiness Levels [16] challenging, when certain conditions are fulfilled, it can effectively support the optimization of lab-scale activities before industrial upscaling [17].

The main challenges concerning the operationalization of SSbD are the integration of circularity into environmental sustainability, data scale-up, and data availability, quality, and uncertainty, which have been rarely addressed in the literature [18]. Therefore, to address this gap and for a more reliable sustainability assessment, a supporting uncertainty study using statistical tools can define a degree of confidence in data gathering, modeling assumptions, and LCA conclusions [19]. Also, to have a more comprehensive view of the issues related to the use of raw materials in innovative processes, a decision-making system should combine studying materials criticality and environmental sustainability, complementary to each other [20]. This integrated approach offers additional dimensions to materials selection and helps prioritize the alternatives by suggesting trade-offs, as discussed in previous works [21,22].

The growing interest in hydrogen separation membranes led to the publication of several recent reports studying the implications of their commercialization. In particular, Martinez et al. [23] performed a systematic LCA analysis on the electroless fabrication of composite Pd membranes to find the environmental hotspots of their lab-scale production, while Alique et al. [24] studied the environmental and economic challenges of implementing different fabrication strategies for such composite membranes. Escalante [25] prepared PdNiAu-based composite membranes via sequential electroless deposition exhibiting high thermal stability and tunable hydrogen permeance depending on the Ni content. Moreover, Sutar [26] proved insightful in highlighting the role of element substitution in the composition of Pd-based membranes on their environmental burdens. This was carried out by comparing the impacts of choosing alternative materials for all three components of electroless-plated membranes.

Recent studies have demonstrated how it is possible to obtain efficient membranes with reduced palladium content via Physical Vapor Deposition (PVD) High-Power Impulse Magnetron Sputtering (HiPIMS) [10,27]. In particular, this peculiar PVD configuration can lead to efficient dense uniform coating of tridimensional shaped substrates, permitting the deposition of metallic layers even onto porous alumina substrates [28,29,30]. Thin Pd films deposited on top of the membrane and the substrate acted as catalytic layers, facilitating the dissociation of hydrogen molecules into atoms, and their subsequent recombination into hydrogen molecules on the opposite layer. This way, the desired hydrogen permeation performance is retained while minimizing the quantity of Pd raw material [31].

Previous works have explored the main drivers of impact at lab-scale preparation of membrane substrates [32] and thin film deposition through PVD magnetron sputtering [33]. Consequently, this study avoids the membrane production processes and focuses its attention only on the choice of raw materials. The aim is to provide insights into the sustainability of materials employed in this field of technology and support future research by proposing a materials selection framework. In this regard, first, the criticality of candidate raw materials used in the composition of layer-based membranes was assessed. Then, the environmental impacts of proposed membrane compositions were compared to those of the reference membrane through LCA analysis, followed by an uncertainty assessment, implementing an updated Ecoinvent database.

## 2. Materials and Methods

### 2.1. System Description

Metallic alloys of different compositions were deposited on porous alumina substrates and tested as hydrogen separation membranes. Candidate compositions were selected based on adequate hydrogen permeation and functional stability [10]. An alloy (Pd_77_Ag_23_) with high Pd content, optimal permeation performance, and resistance to hydrogen embrittlement was considered as the reference membrane [27,34].

**Figure 1:**
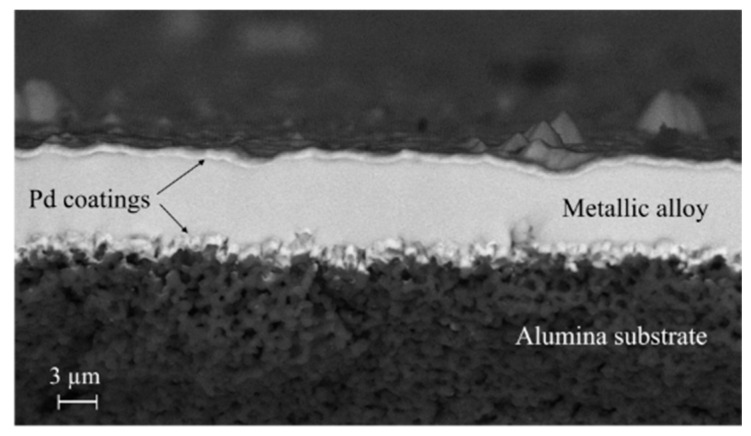
Cross-sectional view of a representative membrane captured under the backscattered electron SEM.

Detailed descriptions of substrate preparation and membrane deposition via HiPIMS are reported in previous works [10,32,35]. Pd coatings (~350 nm) were deposited onto both the surface of the substrate and the dense metallic alloy layer (as seen in Figure 1) to protect the alloys from oxidation, act as catalytic layers, and prevent the interdiffusion phenomena [36]. The Pd thickness was set to neglect its contribution to the permeation of the membranes.

The composition and characteristics of the membranes are listed in Table 1. Due to the high content of Pd, the reference membrane did not require any additional Pd coatings. Selectivity is considered as the ratio between hydrogen and nitrogen permeances, measured at 350–400 °C. It is worth noting that a selectivity value of 400 is actually the limit of sealing system apparatus used for measurements [35].

The microstructure of the crystalline phases of the membranes was studied using X-ray diffraction. Moreover, the surface and cross-section of the samples were observed using Field Emission Scanning Electron Microscopy, while their compositions were determined by Energy Dispersive X-ray Spectroscopy. The permeability of the membranes was measured above 300 °C using a custom-built stainless-steel station [35] and high-purity N_2_ and H_2_ (99.999%), by varying the pressure at the feed side from atmospheric to 400 kPa and maintaining atmospheric pressure on the permeate side. Physicochemical characterization results of the membranes were discussed in previous works [10,27,35].

### 2.2. Critical Raw Materials

The European Commission suggests using Economic Importance (EI) and Supply Risk (SR) criteria to quantify the criticality of raw materials [11]. EI represents vulnerability as a consequence of supply disruption, given the importance of a material in the European Union economy in terms of final applications and added value. SR is the probability that the supply of the material is disrupted, which increases when sourcing is dependent on a dominant provider or a small number of providers (monopolistic supply structure), as opposed to a diversified supplier base.

Another approach to perform an economic-wide criticality assessment is the evaluation of long- and short-term criticality indexes (*Crit._LT_* and *Crit._ST_*) of the raw materials. These criticality indexes were calculated using the data available in the “Resources Scanner” database [37] and through the following equations reported by Bastein & Rietveld [38]:

(1) $$Crit._{LT}=HHI_{res}+P/R+Companionality$$

(2) $$Crit._{ST}=HHI_{prod}\times \left(WGI+OCED\right)\times \left(1-EOL_{RIR}\right)$$

where *HHI* is the Herfindahl–Hirschmann Index, *P/R* is the ratio between production sites and reserves, *Companionality* is the degree to which material is a by-product, *WGI* is the World Governance Indicator, *EoL_RIR_* is the End-of-Life Recycling input rate, and *OCED* indicates the risk of raw materials being affected by export restrictions during the past five years.

### 2.3. Life Cycle Assessment

#### 2.3.1. Goal and Scope

This study investigated how the partial substitution of Pd in the composition of hydrogen separation membranes can affect their environmental footprint. This was performed by comparing the results related to different alloy compositions with diverse Pd contents.

#### 2.3.2. Functional Unit

A preliminary LCA was conducted using a Functional Unit (FU) of 1 kg of each element present in membrane compositions in their refined metal form, according to the European Commission’s suggestion [39].

The second LCA analysis compared the environmental impacts of the studied membrane compositions with those of the reference PdAg membrane. For this comparison, the FU was defined as a thickness of 3.16 µm (equal to the PdAg one), with corresponding LCA reference flow being the mass of each metallic membrane deposited with that thickness onto 2.56 cm^2^ alumina substrate. It is noteworthy that this thickness value falls within a reasonable range for potential implementation in a lab-scale prototype [40].

To account for the performance of the membranes, a further LCA was carried out, using the PdAg membrane permeance as FU. The hypothetical thickness of the membrane required to match the PdAg permeance (2.03 × 10^−6^ mol m^−2^ Pa^−1^ s^−1^ for a 3.16 µm thick membrane) was calculated based on the material permeability. This calculation yielded the corresponding membrane mass reference flow used for the LCA analysis.

#### 2.3.3. System Boundaries and Modeling Assumptions

In this work, the mass of each element present in the metallic layers of the investigated membranes was considered. The actual film production processes and the consumption of resources during the membrane production and characterization steps were neglected. LCA results were discussed as a function of the permeability and gas selectivity of the membranes [32], whilst performance and mechanical stability over time were not considered.

#### 2.3.4. Life Cycle Inventory and Data Sources

Primary data were collected as reported in Section 2.1, and secondary data were obtained from the Econinvent v3.10 database, with adequate temporal representativeness (data from 2011 to 2023), or by adaptations according to similar processes reported in the literature. Specifically, the unit processes for vanadium and zirconium were derived through an adaptation of the titanium unit process (Titanium, triple-melt {GLO} | market for titanium, triple-melt | Cut-off, U), which is available in the Econinvent v3.10 database. This is possible because these three elements are assumed to be produced with the same process, the Kroll method [41,42]. As the pure form of zirconium (sponge) was already present in the Ecoinvent v3.10 database, Zr modelling was limited to the subsequent steps (triple-melt refining and transportation). However, since neither vanadium nor its pure form is included in the database, the entire V life cycle was modelled, including all relevant upstream processes prior to pure element production. A complete inventory of the unit processes used to model the V and Zr flows is provided in Tables S1–S11 of the Supplementary Information (SI).

The selected material flows and their quantities in the composition of membranes are reported in Table 2. The Life Cycle Impact Assessments (LCIAs) were conducted using the SimaPro 9.6 software (PRé sustainability, Amersfoort, The Netherlands), employing the Environmental Footprint (EF) 3.1 method, as recommended by the European Commission [39].

#### 2.3.5. Uncertainty Analysis

All data used in LCA modeling involves some extent of uncertainty that can affect the reliability of the outcome. This is particularly important in the case of comparative LCAs where options are selected based on their impact assessment results. Furthermore, experimental data can contain intrinsic uncertainty, and thus, reporting the uncertainty is essential for transparent communication of results, and to ensure that any future calculations implementing the life cycle inventory will accurately reflect the uncertainty in data [43].

The uncertainties can be distinguished into distinct types, including the correctness of data, variation in data, reliability of measurement means, completeness of the model, and representativeness of the model (considering temporal, geographical, and technological correlations). Addressing uncertain data requires familiarity with data gathering and interpretation of uncertainty specific to the system under study [19].

The Ecoinvent database presents uncertain data with a “best guess” value, together with a range of possible values [44]. This best guess is usually achieved by sampling several measurements and taking the mean of the distribution. A lognormal distribution occurs when values with a normal distribution are multiplied. As this is the case in LCA process chains, lognormal distribution is assumed as the default in the Ecoinvent database [45], and the uncertainty range is represented by the square of the Geometric Standard Deviation (GSD) of a set of measured values [44].

Regarding secondary data, “Pedigree” matrices available in the database were exploited to assess the uncertainty and estimate the GSD of data. The pedigree matrix of each data point consists of score values related to different data quality indicators [44]. In the case of modelled unit processes, the corresponding pedigree matrices were assessed according to uncertainty factors reported in the guidelines [46].

For primary data, key sources of error in data gathering were identified and used to quantify the uncertainty. These include measuring the surface area of the alumina substrates, estimating membrane compositions, and determining film thicknesses.

The assumptions regarding the uncertainty modeling are summarized below:

1. The mass of each membrane (deposited onto 2.56 cm2 alumina substrates) was calculated by considering the mean values of surface area (s), thickness (t), atomic mass (m), and unit cell volume (v). Measuring their respective uncertainty (GSDs, GSDt, GSDm, GSDv) allowed obtaining of the uncertainty of membrane mass [47,48]. The equations regarding the GSD of a set of n values (xi) and error propagation considering the multiplication of parameters are reported below:

(3) $$GSD=\exp\sqrt{1/n\;\sum {\left(\ln x_{i}-\ln{\bar{x}}_{g}\right)}^{2}}\;,\;{\bar{x}}_{g}={\left(\overset{n}{\underset{i=1}{\prod }}x_{i}\right)}^{1/n}$$

(4) $$Mass=surface\;area\times thickness\times \frac{atomic\;mass}{unit\;cell\;volume}$$

(5) $$GSD_{Mass}=\exp\left(\sqrt{{\left[\ln G\;{SD}_{s\;}\;\right]}^{2}+{\left[\ln G\;SD_{t}\;\right]}^{2}+{\left[\ln G\;SD_{m}\;\right]}^{2}+{\left[\ln G\;SD_{v}\;\right]}^{2}}\right)$$

where *x_g_* is the geometric mean.

2. The surface area of several alumina substrates was measured, and the mean and standard deviation were obtained. The contribution of thin film roughness was neglected being lower than the error on thickness estimation, measured using the FE-SEM cross-section images. To assess the coating thickness uniformity, the measurement was repeated in several regions to obtain a distribution of values and their GSD.
3. The composition evaluation of each membrane was carried out by EDS measurements, as reported in a previous work [49], and the corresponding mass GSDs were obtained.
4. The cell parameters of the crystalline phase of the membranes and their errors were acquired by Rietveld refinement of XRD patterns. Assuming an orthorhombic crystalline structure, the unit cell volume and its error were estimated for each membrane.

Finally, the Monte Carlo method was implemented to handle the uncertainty of LCA analysis [50].

## 3. Results and Discussions

### 3.1. CRM Analysis

**Figure 2:**
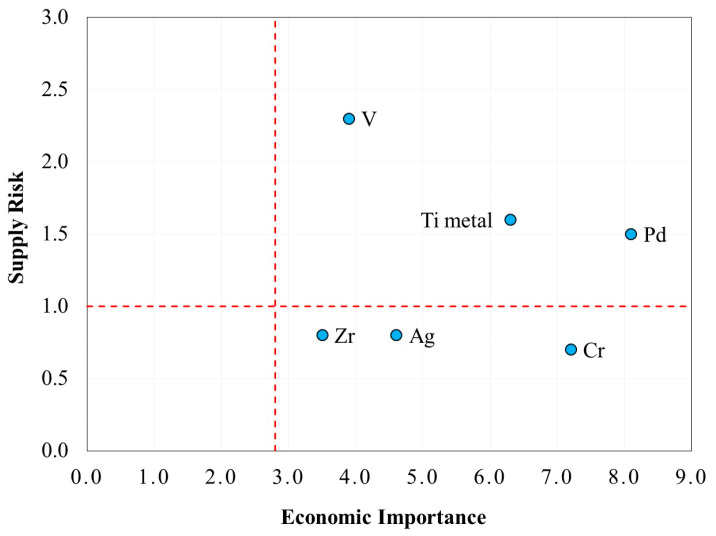
SR and EI indexes for Pd and its potential substitutes. The red dotted lines indicate the criticality threshold values as defined in the 2023 European CRM study (1 for SR, 2.8 for EI) [11].

The criticality indexes of the investigated elements (Pd, V, Ti, Zr, Ag, and Cr) were obtained using the calculations proposed by the European Commission [11]. The results, indicated in Figure 2, show an EI index above the criticality threshold for all the elements: Pd, V and Ti exceeded even the SR index threshold and were, therefore, considered critical.

Compared to the previous European CRM study (2020) [12], the palladium EI index increased substantially due to the change in its estimated added value of the economic sectors. The supply of Platinum Group Metals (PGMs) is strongly dependent on Russia (~1/3 in 2021). Despite the Ukraine–Russia conflict, neither the European Union nor Russia have imposed restrictions on the import or availability of these raw materials. Nevertheless, attempts have been made to increase imports from alternative countries. Progressive risk mitigation is expected due to the drop in demand for Pd in the automotive sector and the development of recovery and recycling processes for PGMs [51].

Regarding vanadium, Europe has been mostly dependent on Russia for its supply (in refined form). Despite major vanadium reserves being in this country, the extraction and refinement activities are carried out mostly in China [11,52]. This anomalous geographical concentration increased the SR index of vanadium in the 2023 CRM update.

In the 2023 European CRM study, titanium metal is considered critical due to its use in high-tech and strategic sectors [53], and the high geographical concentration of suppliers [11].

Studying the long- and short-term criticality of the elements can also be insightful in managing the risk associated with the raw materials supply chain. In this regard, these criticality indexes were calculated as described in Section 2.2 [38]. The value of each criticality parameter according to the updated database (2022) is reported in Table 3.

It should be noted that high P/R indicates high risks from proven reserves which can lead to an increase in the long-term criticality of materials. For Pd, the geographical location of reserves and the high degree of specialization required led to its mining and refining activities to be concentrated in a few countries, namely Russia, South Africa, and Canada. Moreover, it can be concluded that materials with a high recycling rate have low short-term criticality (e.g., Cr with null criticality), while the ones with inadequate recycling and a high concentration in countries with poor qualities of governance (high WGI) or restrictive export policies (high OCED) tend to have greater short-term criticality (e.g., V).

**Figure 3:**
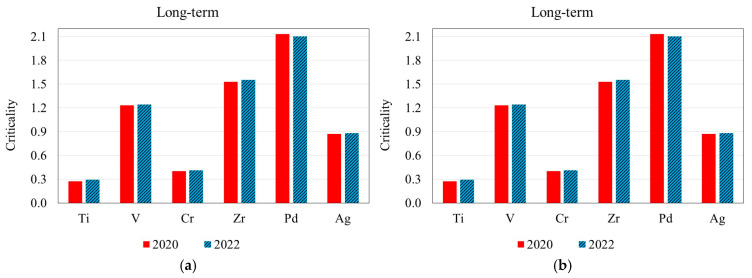
Comparison of (**a**) short-term and (**b**) long-term criticality values, according to indexes of 2020 and 2022.

A comparison between the 2020 and the 2022 criticality indexes of each element is illustrated in Figure 3.

As can be observed from the graphs, except for the particular case of Cr, the data published in 2022 showed a decrease in the WGI index for all elements, and a marked decrease for V, Zr, and Pd, leading to a decrease in the Crit._ST_ index. On the other hand, Crit._LT_ index did not show any significant variations.

### 3.2. LCA Results

**Figure 4:**
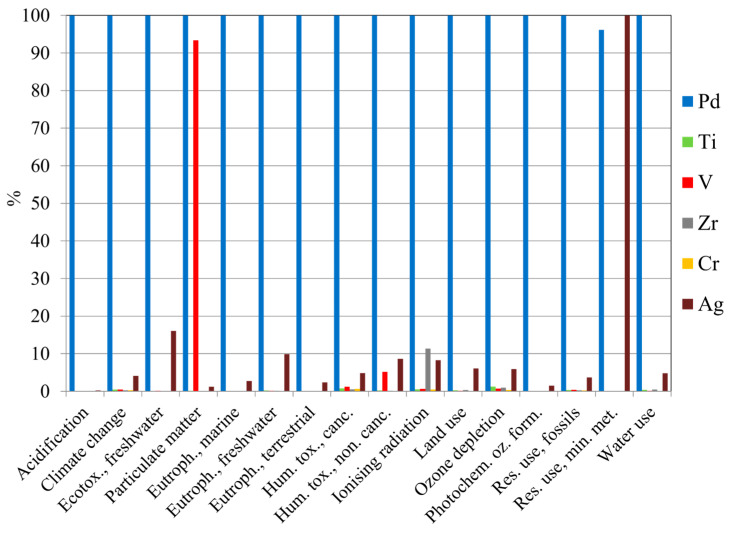
Characterized results of the investigated elements to each environmental impact category (FU: 1 kg of each refined metal present in the membrane compositions).

A first assessment was performed considering 1 kg of each element present in the membrane compositions, in their refined metal form, as FU. Also, an uncertainty analysis was carried out by incorporating the corresponding pedigree matrices associated with the production of each element, as provided in the Ecoinvent 3.10 database. Figure 4 illustrates a comparison of the characterized results of the investigated elements in each environmental impact category (listed in Table S12 of SI). A list of the main environmental impact categories, their units of measurement, their abbreviations and descriptions are reported in Table S13.

The results indicated that palladium had a significant contribution across almost all impact categories, except in “Resource use, minerals and metals” where silver had the highest impact. In addition, vanadium showed a notable impact in the “Particulate Matter” category.

**Figure 5:**
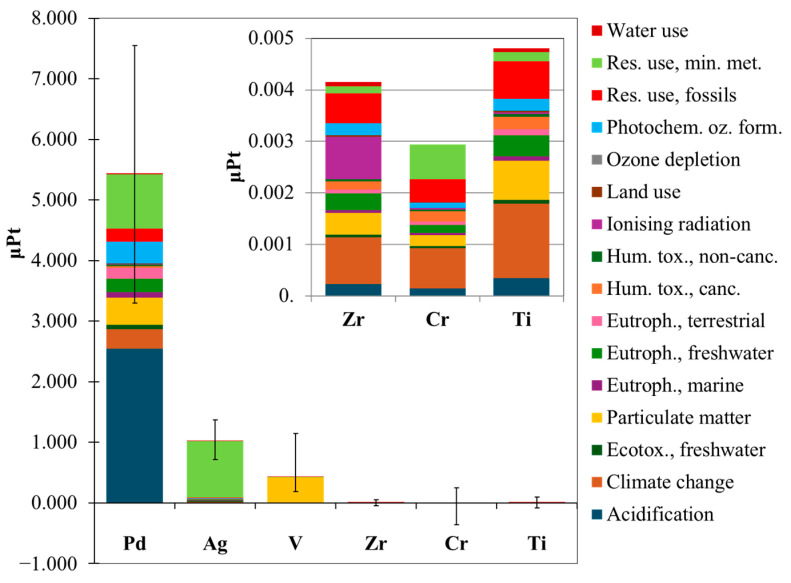
Single score results of the elements studied. The dimensionless unit µPt stands for micropoints (FU: 1 kg of each refined metal present in the membrane compositions).

By normalizing and weighing the characterization results, it is possible to add the contribution of different impact categories, forming a “single score” index, and obtaining an overall view of various environmental burdens related to each element within their uncertainty range (100 Monte Carlo simulations and 95% confidence interval), as shown in Figure 5.

Figure 5 highlights the distinct contribution of each element to various impact categories. It is evident that Pd, Ag, and V exhibit relatively high environmental burdens, with Pd single score being 5.3 and 12.8 times larger than Ag and V, respectively. The most impacted categories were “Acidification” and “Resource use, minerals and metals”. This simplified analysis highlights how Pd has a significant potential environmental impact, and the objective of its replacement in membrane compositions is of utmost importance to make hydrogen separation technology more sustainable.

After identifying the environmental burdens of each element, LCA and uncertainty analysis were performed on investigated membrane compositions. To have a representative comparison, the environmental impacts of these membranes were determined by imposing the same value of thickness on all the membranes, equal to the reference (3.16 µm), and using a consistent surface area for the alumina substrates (2.56 cm^2^). With these parameters fixed, and knowing the density of the crystalline phase of each membrane (obtained from the XRD analyses), the corresponding mass value and its uncertainty (see Section 2.3.5) were calculated for each membrane, as reported in Table S14 of SI.

**Figure 6:**
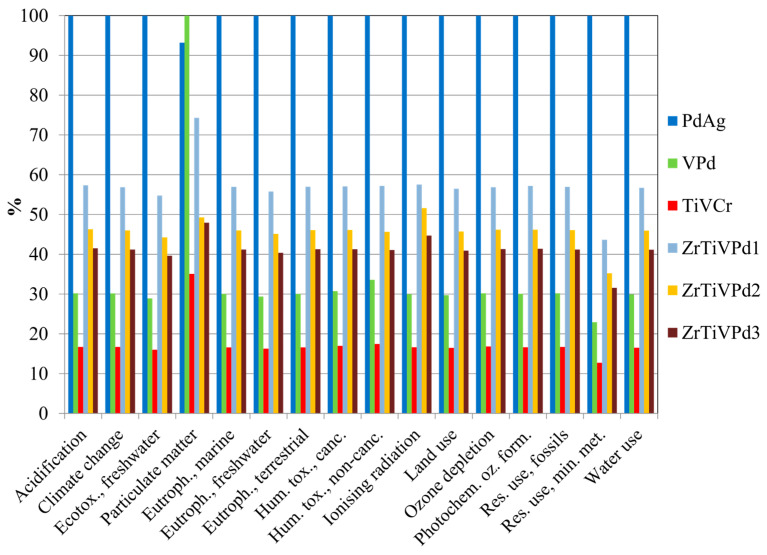
Characterized results of the studied membranes. FU: thickness of 3.16 µm (equal to the PdAg one); the LCA reference flow was the mass of each metallic membrane deposited with that thickness onto 2.56 cm^2^ alumina substrate.

The LCIA characterization results of the studied membranes are shown in Figure 6, while Table S15 of the SI lists characterization results and their associated uncertainties. As expected, the PdAg membrane exhibited the highest characterized results across all impact categories, except for “Particulate Matter” where the VPd membrane showed the highest contribution. Analyzing the uncertainty of impact values across different categories for all the membranes, “Water use” and “Human toxicity, non-cancer” showed the largest coefficient of variability, indicating the highest uncertainty.

**Figure 7:**
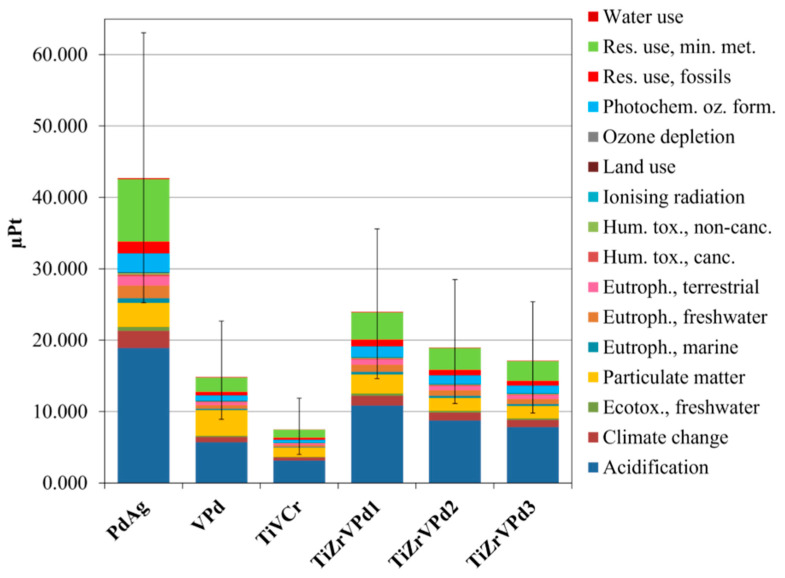
Single score results of the studied membranes. FU: thickness of 3.16 µm (equal to the PdAg one); the LCA reference flow was the mass of each metallic membrane deposited with that thickness onto 2.56 cm^2^ alumina substrate.

After normalizing and weighing the characterization results, the single score outcomes of the membranes were obtained, as shown in Figure 7, together with their respective uncertainty range, considering a 95% confidence interval.

Compared to the reference membrane, which showed the highest overall impact, the least impacting membrane was the TiVCr (82.5% lower), followed by the VPd (65.3% lower). Furthermore, it is evident that reducing the Pd content in TiZrVPd membranes effectively lowered the weighted environmental impacts, and on average, their overall impact is 53.1% lower than the PdAg membrane. The impact categories with the highest contribution to the overall impacts were found to be “Acidification”, “Resource use, minerals and metals”, and “Particulate matter” for all the investigated membranes.

To address the uncertainty in LCA results, the number of Monte Carlo iterations can be limited by manually setting a maximum number of runs or defining a fixed Standard Error of Mean. To study the effect of the number of calculations, the simulations were repeated for 100, 1000, and 10,000 runs. However, little to no change was observed in the resulting single score values and their corresponding standard deviations, indicating a statistically stable model that is convergent even at a small number of simulations (100). The results of this investigation are reported in Table S16 and Figure S1 of SI.

To account for membrane performance when comparing the environmental burdens, the membranes were evaluated by considering their hydrogen permeability and gas selectivity at 350 °C. Permeability is a material property while permeance is an absolute indicator depending on the thickness. Also, selectivity is considered as the ratio between H_2_ and N_2_ permeance [10]. It is important to note that TiVCr and TiZrVPd membranes possessed low selectivity values compared to the PdAg membranes. Despite exhibiting permeability values close to the reference (6.41 × 10^−12^ mol m^−1^ Pa^−1^ s^−1^) [10], low selectivity ultimately makes these membranes practically unsuitable for the application of hydrogen separation.

**Figure 8:**
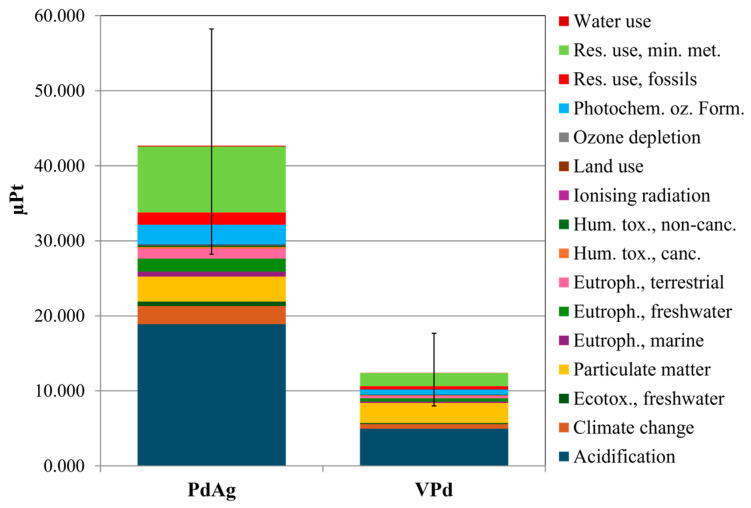
Single score results of the VPd and PdAg membranes and their respective uncertainty ranges, considering 95% confidence interval. FU: the PdAg membrane permeance (2.03 × 10^−6^ mol m^−2^ Pa^−1^ s^−1^ for a 3.16 µm thick membrane); the LCA reference flow of PdV was the mass of the membrane deposited with a thickness of 2.66 μm onto 2.56 cm^2^ alumina substrate.

On the contrary, VPd with a high selectivity (80% higher than the reference), a permeability of 5.40 × 10^−12^ mol m^−1^ Pa^−1^ s^−1^, and lower total impacts than the PdAg reference membrane, can be considered as a suitable candidate composition for such membranes. To account for the performance of the membranes, a further LCA was carried out, using the PdAg membrane permeance as FU. The hypothetical thickness of the VPd membrane required to match the PdAg permeance (2.03 × 10^−6^ mol m^−2^ Pa^−1^ s^−1^ for a 3.16 µm thick membrane) was calculated based on the material permeability. This calculation yielded the corresponding membrane mass reference flow used for the LCA analysis. The resulting thickness value was 2.66 μm, which remained reasonable for a potential practical application. Therefore, the reference flow (i.e., the respective membrane masses) used to carry out the LCA analysis was calculated as described previously in this section. As is possible to observe in Figure 8, the whole impact burden of VPd (the LCA characterized results are listed in Table S17 of SI) resulted to be 71% lower than the PdAg.

**Figure 9:**
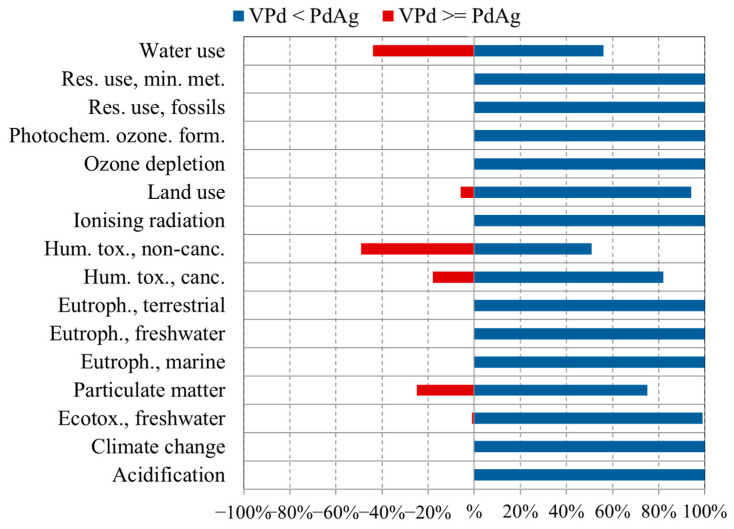
Comparison of the characterized impact results between VPd and PdAg membranes for each impact category, derived from 100 Monte Carlo simulations. FU: permeability of the membranes, whilst their masses (considering the permeance and an area of 2.56 cm^2^) was used as LCA reference flow.

Discernability is an approach to analyze the uncertainty in comparative LCAs, supporting a more detailed comparison of the options [54]. Considering the above mentioned LCA, Figure 9 indicates the frequency of Monte Carlo outcomes where one membrane has a higher impact score than the other, per impact category. In case of significant differences (100% positive or negative), options can be compared with less uncertainty, and high data quality can be assumed. Otherwise, uncertainty is considered high.

Figure 9 illustrates that, in comparison, the PdAg membrane has consistently higher values across all impact categories except for five, underlining its relatively greater environmental burdens. As an example, it is evident that PdAg showed a higher impact in the “Human toxicity, cancer” category in 81% of runs; meaning that in this category, PdAg has a higher value than VPd with 81% certainty.

Currently, LCA can solely evaluate resource use under the “Resource depletion” category, focusing on the effect of raw materials extraction. This method has limited comprehensiveness in representing resources as few CRMs are modeled in its databases. Also, the security of the supply of resources and the socio-economic factors that affect raw material access are not explicitly considered in current impact assessment methods. The development of LCA methodology can offer strategies for not only assessing the environmental implications of materials but also establishing proper indicators for CRM identification [55].

## 4. Conclusions

This study provides a methodological approach to support materials selection for sustainable hydrogen separation membranes. Several raw materials with promising hydrogen permeation and low criticality were investigated, and novel membrane compositions with reduced Pd content were compared based on their permeability and environmental impacts. The CRM analysis highlighted the current criticality status of each element. A metal criticality assessment is regarded as a dynamic study that should be periodically updated to account for the alterations in the supply chain. It was shown that the inconvenient geographical concentration of Pd reserves has resulted in its long-term criticality, highlighting the need for alternatives. The LCA of producing the raw materials used in membrane compositions showed that Pd, Ag, and V exhibit relatively high environmental burdens, with a Pd single score 5.3 and 12.8 times larger than Ag and V, respectively. Comparative LCA of the membranes indicated that the ZrVTiPd membranes display 53.1% lower total impacts than those of the PdAg reference membrane on average, and the partial substitution of Pd in their composition substantially reduced the overall impacts.

Moreover, the VPd membrane can be considered a suitable candidate, having lower environmental impacts than the reference membrane (65% or 71% considering thickness of 3.16 µm or permeance of 2.03 × 10^−6^ mol m^−2^ Pa^−1^ s^−1^, respectively), showing a higher gas selectivity feature. While the literature tends to overlook the practical challenges associated with managing the life cycle inventories, this work tried to address this gap by studying the uncertainty in data gathering and the way it affects the accuracy of the outcome. Monte Carlo analysis proved useful in indicating the distribution of impact values, highlighting the extent of uncertainty in results, and pairwise comparison of membranes at different impact categories, without altering the principal conclusions of the LCIAs. It can be concluded that a broad sustainability analysis implementing LCA thinking would be beneficial for a comprehensive assessment of risks associated with various aspects of a product lifecycle and supply chain.

## Figures and Tables

**Table 1:** Composition of the metallic layer of the studied membranes and their features.

Membrane	Composition	Thickness (μm)	Alloy Pd Content (wt%)	Permeance (mol m^−2^ Pa^−1^ s^−1^)	Selectivity
TiVCr	Ti_20_V_33_Cr_47_	2.0 ± 0.2	-	1.4 × 10^−6^	11
VPd [35]	V_93_Pd_7_	3.5 ± 0.2	14%	1.5 × 10^−6^	400
PdAg [27]	Pd_77_Ag_23_	3.2 ± 0.2	65%	2.0 × 10^−6^	222
ZrVTiPd1 [10]	Zr_9_V_34_Ti_30_Pd_27_	4.7 ± 0.2	37%	3.5 × 10^−6^	50
ZrVTiPd2 [10]	Zr_52_V_12_Ti_13_Pd_23_	6.1 ± 0.2	36%	3.5 × 10^−6^	100
ZrVTiPd3 [10]	Zr_39_V_20_Ti_19_Pd_22_	6.4 ± 0.2	33%	8.1 × 10^−6^	75

**Table 2:** Unit processes and corresponding quantities (in kg) of the elements used in the studied membranes.

Element/Ecoinvent Process		Pd_77_Ag_23_	V_93_Pd_7_	Ti_20_V_33_Cr_47_	ZrVTiPd1	ZrVTiPd2	ZrVTiPd3
Pd	Palladium {GLO} | market for palladium| Cut-off, U	0.768	0.136	-	0.419	0.291	0.299
Ag	Silver {GLO} | market for silver | Cut-off, U	0.232	-	-	-	-	-
Cr	Chromium {GLO} | market for chromium | Cut-off, U	-	-	0.481	-	-	-
Zr	Zirconium triple-melt (modelled)	-	-	-	0.120	0.563	0.455
Ti	Titanium, triple-melt {GLO}| market for titanium, triple-melt | Cut-off, U	-	-	0.188	0.209	0.073	0.116
V	Vanadium triple-melt (modelled)	-	0.864	0.331	0.252	0.073	0.130

**Table 3:** Parameters and calculation of the short-term and long-term criticality for Pd and its potential substitutes according to the 2022 indexes [37] (accessed on 19 June 2025).

Element	P/R	Comp.	HHI_res_	Crit._LT_	HHI_prod_	WGI	OECD	EOL_RIR_	Crit._ST_
Pd	0.16	0.97	0.97	2.100	0.34	0.2	0.00	0.22	0.053
Ag	0.02	0.72	0.14	0.880	0.1	0.08	0.00	0.75	0.002
Cr	0.15	0.02	0.24	0.410	0.22	0.18	0.02	1.00	0.000
Zr	0.03	1.00	0.52	1.550	0.21	0.28	0.00	0.06	0.055
Ti	0.10	0.00	0.19	0.290	0.11	0.16	0.00	0.33	0.012
V	0.18	0.74	0.32	1.240	0.36	0.28	0.53	0.01	0.289

## Data Availability

The raw data supporting the conclusions of this article will be made available by the authors on request.

## Supplementary Materials

The following supporting information can be downloaded at: https://www.mdpi.com/article/10.3390/membranes15100310/s1, Table S1: Zirconium production unit process inventory. This process was adapted from the “Titanium, triple-melt {GLO}| titanium production, triple-melt | Cut-off, U” unit process, with electricity consumption according to Norgate et al. [1]. As the unit process for zirconium sponge production is already present in the Ecoinvent 3.10 database, modelling the zirconium market unit process was carried out only by considering the triple-melt process and transportation, according to the titanium market unit process, Table S2: Zirconium market unit process inventory. This is the market for “Zirconium triple-melt production | Cut-off, U”, in the global geography (GLO), adapted from “Titanium, triple-melt {GLO}| market for titanium, triple-melt”, Table S3: Vanadium bearing magnetite (Magnetite 72% Fe, 2.2% V_2_O_5_) production unit process inventory. As the unit process for vanadium or its sponge is not present in the Ecoinvent 3.10 database, the entire model was built manually, according to the titanium triple-melt production processes available in the database, Table S4: Pre-reduced V_2_O_5_ magnetite production unit process inventory. This process was adapted from “Iron pellet {RoW}| iron pellet production | Cut-off, U”, Table S5: Vanadium Slag (25% V_2_O_5_) production unit process inventory, Table S6: Vanadium Pentaoxide (V_2_O_5_) production unit process inventory, Table S7: Vanadium chloride (VCl_3_) production unit process inventory [3], Table S8: Vanadium unrefined production unit process inventory. This process was adapted from “Titanium sponge {RoW}| titanium sponge production, from titanium tetrachloride | Cut-off, U” [2], Table S9: Vanadium unrefined market unit process inventory. This is the market for vanadium unrefined production and was adapted from “Titanium sponge {GLO}| market for titanium sponge | Cut-off, U”, Table S10: Vanadium production unit process inventory. This process was adapted from titanium triple-melt production and represents the melting purification process [1], Table S11: Vanadium market unit process inventory. This is the market for “Vanadium, triple-melt production” was adapted from “Titanium, triple-melt {GLO}| market for titanium, triple-melt”, Table S12: LCA characterization results (EF 3.1 method) and uncertainty (100 simulations) with the FU of 1 kg of each refined metal present in the membrane compositions (95% confidence interval), Table S13: List of main environmental impact categories, their units of measurement and descriptions, gathered from the EN15804 standard and the Ecoinvent 3.10 database, Table S14: LCA inventory of the studied membranes. FU is thickness of 3.16 µm (equal to the PdAg one), and the LCA reference flow was the mass of each metallic membrane deposited with that thickness onto 2.56 cm^2^ alumina substrate, Table S15: LCA characterized results of the studied membranes, based on the Pd_77_Ag_23_ thickness (EF 3.1 method, and 95% confidence interval). The FU is thickness of 3.16 µm (equal to the PdAg one), and the LCA reference flow was the mass of each metallic membrane deposited with that thickness onto 2.56 cm^2^ alumina substrate, Table S16: Uncertainty analysis results for varying numbers of Monte Carlo simulations (95% confidence interval), comparison of the single score results of the studied membranes and their uncertainty range. The FU is thickness of 3.16 µm (equal to the PdAg one), and the LCA reference flow was the mass of each metallic membrane deposited with that thickness onto 2.56 cm^2^ alumina substrate. The unit is µPt, Table S17: LCA characterization results of comparing the VPd and PdAg membranes The functional unit is the PdAg membrane permeance (2.03×10^−6^ mol m^−2^ Pa^−1^ s^−1^ for a 3.16 µm thick membrane), and the LCA reference flow of PdV was the mass of the membrane deposited with a thickness of 2.66 μm onto 2.56 cm^2^ alumina substrate, Figure S1: Comparison of the single score results (EF 3.1 method) and the uncertainty range of the studied membranes after different numbers of Monte Carlo calculations. FU: thickness of 3.16 µm (equal to the PdAg one) and the LCA reference flow was the mass of each metallic membrane deposited with that thickness onto 2.56 cm^2^ alumina substrate). References [42,56,57] are cited in the supplementary materials.

## Abbreviations

The following abbreviations are used in this manuscript:

CRMs	Critical raw materials
SSbD	Safe and Sustainable by Design
LCA	Life Cycle Assessment
LCIA	Life Cycle Impact Assessment
XRD	X-ray Diffraction
EDS	Energy Dispersive X-ray Spectroscopy
FE-SEM	Field Emission Scanning Electron Microscopy
EI	Economic Importance
SR	Supply Risk
EF	Environmental Footprint
GLO	Global
GSD	Geometric Standard Deviation
HiPIMS	High-Power Impulse Magnetron Sputtering
PGMs	Platinum Group Metals
