# Supplementary material for: Life Cycle Assessment and Critical Raw Materials Analysis of Innovative Palladium-Substituted Membranes for Hydrogen Separation
Source: Membranes (Basel). 2025 Oct 13;15(10):310. doi: 10.3390/membranes15100310 (PMC12565921; doi:10.3390/membranes15100310)
Supplement: Supplementary file 1 [file membranes-15-00310-s001.zip › membranes-3896891-supplementary.pdf]

membranes

MDPI

Supplementary Materials

Life Cycle Assessment and Critical Raw Materials Analysis of Innovative Palladium-Substituted Membranes for Hydrogen Separation

Ali Mohtashamifar, Simone Battiston *, Stefano Fasolin, Stefania Fiameni, Francesca Visentin and Simona Barison

Institute of Condensed Matter Chemistry and Technologies for Energy (ICMATE), National Research Council of Italy (CNR), C.So Stati Uniti 4, 35127 Padua, Italy; alimohtashamifar@cnr.it (A.M.); stefano.fasolin@cnr.it (S.F.); stefania.fiameni@cnr.it (S.F.); francesca.visentin@cnr.it (F.V.); simona.barison@cnr.it (S.B.)

* Correspondence: simone.battiston@cnr.it

In this file, supplementary materials that are related to and mentioned in the main manuscript are reported. It includes life cycle inventories, a list of main environmental impact categories, impact assessment results, and uncertainty results with respective descriptions.

Abbreviations

**GSD**	Geometric Standard Deviation
**SD**	Standard Deviation
**na**	Not Available
**RoW**	Rest of the World
**RER**	Europe
**U**	Unit
**CV**	Coefficient of Variability
**p**	Piece
**SEM**	Standard Error of Mean

Table S1. Zirconium production unit process inventory. This process was adapted from the “Titanium, triple-melt {GLO}| titanium production, triple-melt | Cut-off, U” unit process, with electricity consumption according to Norgate et al. [1]. As the unit process for zirconium sponge production is already present in the Ecoinvent 3.10 database, modelling the zirconium market unit process was carried out only by considering the triple-melt process and transportation, according to the titanium market unit process.

**Output**	**Amount**	**Unit**	**Distribution**	**SD**	**Comments**
Zirconium triple-melt production	1.000	kg			
**Input**					
Zirconium sponge, nuclear-grade {GLO}| market for zirconium sponge, nuclear-grade	Cut-off, U	1.000	kg	Lognormal	2.504	(5,5,5,5,5,na)* This matrix was manually inserted.
Electricity, high voltage {GLO}| market group for electricity, high voltage	Cut-off, U	4.000	kW	Lognormal	1.492	(1,1,5,1,1,na)

*pedigree matrix

Table S2. Zirconium market unit process inventory. This is the market for "Zirconium triple-melt production | Cut-off, U", in the global geography (GLO), adapted from "Titanium, triple-melt {GLO}| market for titanium, triple-melt". .

Output	Amount	Unit	Distribution	SD	Comments
Zirconium triple-melt	1.000	kg			
**Input**					
Zirconium triple-melt production	1.000	kg	Lognormal	1.890	(4,5,5,5,4,na) This matrix was manually inserted.
Transport, freight train {GLO}| market group for transport, freight train	Cut-off, U	0.345	tkm	Lognormal	2.248	(1,1,3,5,4,na)
Transport, freight, inland waterways, barge {GLO}| market group for transport, freight, inland waterways, barge	Cut-off, U	0.036	tkm	Lognormal	2.248	(1,1,3,5,4,na)
Transport, freight, lorry, unspecified {GLO}| market group for transport, freight, lorry, unspecified	Cut-off, U	0.361	tkm	Lognormal	2.248	(1,1,3,5,4,na)
Transport, freight, sea, container ship {GLO}| market for transport, freight, sea, container ship	Cut-off, U	0.363	tkm	Lognormal	2.248	(1,1,3,5,4,na)

Table S3. Vanadium bearing magnetite (Magnetite 72% Fe, 2.2% V_2O_5) production unit process inventory. As the unit process for vanadium or its sponge is not present in the Ecoinvent 3.10 database, the entire model was built manually, according to the titanium triple-melt production processes available in the database.

Output	Amount	Unit	Distribution	SD	Comments
Vanadium bearing magnetite (Magnetite 72% Fe, 2.2% V_2O_5)	1.530	kg	undefined		Main product (83%)
Ilmenite, 54% titanium dioxide	0.460	kg	undefined		Co-product (17%)
Particulates, < 2.5 um	1.800×10^{-5}	kg	undefined		
Particulates, < 10 um	2.400×10^{-4}	kg	undefined		
Particulates, > 2.5 um, and < 10um	9.600×10^{-5}	kg	undefined		
Water	0.008	kg	undefined		
Water, GLO	5.000×10^{-5}	m^3	undefined		
			undefined		
**Input**			undefined		
Occupation, mineral extraction site	0.002	m^2a	undefined		
Titanium oxide	0.250	kg	undefined		
Iron ore	1.080	kg	undefined		
Transformation, from forest, unspecified	8.300×10^{-5}	m^2	undefined		
Transformation, to mineral extraction site	8.300×10^{-5}	m^2	undefined		
Vanadium ore	0.034	kg	undefined		

Water, well, GLO	5.900×10^{-5}	m^3	undefined		
Blasting {GLO}∣ market for blasting ∣ Cut-off, U	1.500×10^{-4}	kg	undefined		
Conveyor belt {GLO}∣ market for conveyor belt ∣ Cut-off, U	5.600×10^{-8}	m	undefined		
Diesel, burned in diesel–electric generating set, 10MW {GLO}∣ market for diesel, burned in diesel–electric generating set, 10MW ∣ Cut-off, U	0.024	MJ	undefined		
Electricity, medium voltage {CN}∣ market group for electricity, medium voltage ∣ Cut-off, U	0.014	kWh	undefined		
Heat, central or small-scale, natural gas {GLO}∣ market group for heat, central or small-scale, natural gas ∣ Cut-off, U	0.015	MJ	undefined		
Industrial machine, heavy, unspecified {RoW}∣ market for industrial machine, heavy, unspecified ∣ Cut-off, U	1.200×10^{-5}	kg	undefined		
Mine infrastructure, open cast, ilmenite from hard-rock ore {GLO}∣ market for mine infrastructure, open cast, ilmenite from hard-rock ore ∣ Cut-off, U	3.100×10^{-11}	p	undefined		
Petrol, unleaded {RoW}∣ market for petrol, unleaded ∣ Cut-off, U	0.063	kg	undefined		
Recultivation, ilmenite mine {GLO}∣ market for recultivation, ilmenite mine ∣ Cut-off, U	8.300×10^{-5}	m^2	undefined		
Particulates, < 2.5 um	1.800×10^{-5}	kg	undefined		
Particulates, < 10 um	2.400×10^{-4}	kg	undefined		
Particulates, > 2.5 um, and < 10um	9.600×10^{-5}	kg	undefined		
Water	8.790×10^{-3}	kg	undefined		
Water, GLO	5.000×10^{-5}	m^3	undefined		

Table S4. Pre-reduced V_2O_5 magnetite production unit process inventory. This process was adapted from "Iron pellet {RoW}∣ iron pellet production ∣ Cut-off, U".

**Output**	**Amount**	**Unit**	**Distribution**	**SD**	Comments
Pre-reduced V_2O_5 magnetite production	1.000	kg			adapted from Iron pellet {RoW}∣
Cadmium (II)	2.100×10^{-10}	kg	Lognormal	5.015	(2,3,5,3,1,na)
Carbon dioxide, fossil	0.024	kg	Lognormal	1.514	(2,3,5,3,1,na)
Carbon dioxide, fossil (low. pop.)	7.583×10^{-4}	kg	Lognormal	1.050	(2,2,5,5,1,na)
Carbon monoxide, fossil	2.100×10^{-4}	kg	Lognormal	5.015	(2,3,5,3,1,na)

Carbon monoxide, fossil (low. pop.)	2.089 × 10^{-8}	kg	Lognormal	5.015	(2,2,5,5,1,na)
Chromium, ion	2.700 × 10^{-9}	kg	Lognormal	5.015	(2,3,5,3,1,na)
Copper, ion	4.600 × 10^{-9}	kg	Lognormal	5.015	(2,3,5,3,1,na)
Dinitrogen monoxide (low. pop.)	1.497 × 10^{-9}	kg	Lognormal	1.491	(2,2,5,5,1,na)
Dioxin, 2,3,7,8 Tetrachlorodibenzo-p-	5.700 × 10^{-15}	kg	Lognormal	1.491	(2,3,5,3,1,na)
Hydrocarbons, aliphatic, alkanes, unspecified	2.250 × 10^{-5}	kg	Lognormal	2.990	(2,3,5,3,1,na)
Hydrochloric acid	2.500 × 10^{-5}	kg	Lognormal	1.491	(2,3,5,3,1,na)
Hydrochloric acid (low. pop.)	1.979 × 10^{-7}	kg	Lognormal	1.491	(3,2,5,5,1,na)
Hydrogen fluoride	1.990 × 10^{-5}	kg	Lognormal	1.491	(2,3,5,3,1,na)
Hydrogen fluoride (low. pop.)	4.631 × 10^{-7}	kg	Lognormal	1.491	(3,2,5,5,1,na)
Lead (II)	6.650 × 10^{-8}	kg	Lognormal	1.491	(2,3,5,3,1,na)
Manganese (II)	2.300 × 10^{-8}	kg	Lognormal	5.015	(2,3,5,3,1,na)
Mercury (II)	2.500 × 10^{-10}	kg	Lognormal	5.015	(2,3,5,3,1,na)
Mercury (II) (low. pop.)	9.882 × 10^{-11}	kg	Lognormal	5.015	(3,2,5,5,1,na)
Methane, fossil (low. pop.)	6.09899 × 10^{-11}	kg	Lognormal	5.015	(2,2,5,5,1,na)
Nickel (II)	0.000000015	kg	Lognormal	1.491	(2,3,5,3,1,na)
Nitrogen oxides	0.000315	kg	Lognormal	5.015	(2,3,5,3,1,na)
Nitrogen oxides (low. pop.)	1.964 × 10^{-7}	kg	Lognormal	1.491	(2,2,5,5,1,na)
NMVOCs, non-methane volatile organic compounds (low. pop.)	4.197 × 10^{-11}	kg	Lognormal	1.491	(2,2,5,5,1,na)
PAHs, polycyclic aromatic hydrocarbons	1.900 × 10^{-10}	kg	Lognormal	1.491	(2,3,5,3,1,na)
Particulates, < 2.5 um	7.500 × 10^{-5}	kg	Lognormal	2.990	(2,3,5,3,1,na)
Particulates, < 2.5 um (low. pop.)	3.388 × 10^{-7}	kg	Lognormal	2.990	(2,2,5,5,1,na)
Particulates, > 10 um (low. pop.)	5.857 × 10^{-7}	kg	Lognormal	2.990	(2,2,5,5,1,na)
Particulates, > 2.5 um, and < 10um (low. pop.)	4.015 × 10^{-7}	kg	Lognormal	1.492	(2,2,5,5,1,na)
Silver (I) (low. pop.)	5.642 × 10^{-11}	kg	Lognormal	1.999	(3,2,5,5,1,na)
Sulfur dioxide	1.340 × 10^{-4}	kg	Lognormal	5.015	(2,3,5,3,1,na)
Sulfur dioxide (low. pop.)	9.396 × 10^{-7}	kg	Lognormal	1.514	(2,2,5,5,1,na)
Vanadium	8.550 × 10^{-8}	kg	Lognormal	1.050	(2,3,5,3,1,na)
Water/m3	3.246 × 10^{-6}	kg	Lognormal	5.015	(2,2,5,1,1,na)
Zinc (II)	5.620 × 10^{-8}	kg	Lognormal	1.492	(2,3,5,3,1,na)
Arsenic, ion (river)	6.203 × 10^{-12}	kg	Lognormal	5.015	(2,2,5,5,1,na)
Cadmium (II) (river)	3.101 × 10^{-12}	kg	Lognormal	5.015	(2,2,5,5,1,na)
Chromium, ion (river)	4.961 × 10^{-11}	kg	Lognormal	5.015	(2,2,5,5,1,na)
Cobalt (river)	6.203 × 10^{-12}	kg	Lognormal	5.015	(2,2,5,5,1,na)
Copper, ion (river)	6.203 × 10^{-12}	kg	Lognormal	5.015	(2,2,5,5,1,na)
Iron, ion (river)	1.179 × 10^{-8}	kg	Lognormal	5.015	(2,2,5,5,1,na)
Lead (II) (river)	6.203 × 10^{-12}	kg	Lognormal	5.015	(2,2,5,5,1,na)

Manganese (II) (river)	1.861×10^{-9}	kg	Lognormal	5.015	(2,2,5,5,1,na)
Mercury (II) (river)	8.062×10^{-13}	kg	Lognormal	5.015	(2,2,5,5,1,na)
Nickel (II) (river)	6.203×10^{-12}	kg	Lognormal	5.015	(2,2,5,5,1,na)
Nitrate (river)	1.141×10^{-8}	kg	Lognormal	5.015	(2,2,5,5,1,na)
Phosphorus (river)	7.443×10^{-10}	kg	Lognormal	5.015	(2,2,5,5,1,na)
Suspended solids, unspecified (river)	3.972×10^{-8}	kg	Lognormal	5.015	(2,2,5,5,1,na)
Water, RoW	1.839×10^{-5}	kg	Lognormal	5.015	(2,2,5,1,1,na)
Zinc (II) (river)	2.481×10^{-11}	kg	Lognormal	5.015	(2,2,5,5,1,na)
Scrap steel {CH}| market for scrap steel | Cut-off, U	4.116×10^{-9}	kg	Lognormal		(1,2,5,5,1,na)
Scrap steel {Europe without Switzerland}| market for scrap steel | Cut-off, U	3.256×10^{-6}	kg	Lognormal	5.015	(1,2,5,5,1,na)
Spent solvent mixture {Europe without Switzerland}| market for spent solvent mixture | Cut-off, U	1.230×10^{-7}	kg	Lognormal	5.015	(1,2,5,5,1,na)
Waste mineral oil {Europe without Switzerland}| market for waste mineral oil | Cut-off, U	8.660×10^{-8}	kg	Lognormal	5.015	(1,2,5,5,1,na)
Wastewater, average {Europe without Switzerland}| market for wastewater, average | Cut-off, U	0.007	m^3	undefined		
Input					
Water, unspecified natural origin, RoW	9.000×10^{-5}	m^3	Lognormal	1.505	(1,2,5,1,1,na)
Aluminium oxide factory {GLO}| market for aluminium oxide factory | Cut-off, U	2.500×10^{-11}	p	Lognormal	4.647	(5,5,5,5,5,na)
Chromium {GLO}| market for chromium | Cut-off, U	5.968×10^{-6}	kg	Lognormal	1.504	(1,1,5,1,1,na)
Dolomite {RER}| market for dolomite | Cut-off, U	0.001	kg	Lognormal	1.050	(1,2,5,5,1,na)
Hard coal {CN}| market for hard coal | Cut-off, U	0.013	kg	Lognormal	1.506	(1,2,5,3,1,na)
Lime {RER}| market for lime | Cut-off, U	5.701×10^{-4}	kg	Lognormal	1.050	(1,2,5,5,1,na)
Lubricating oil {RER}| market for lubricating oil | Cut-off, U	4.602×10^{-7}	kg	Lognormal	1.504	(1,1,5,1,1,na)
Sodium hydroxide, without water, in 50% solution state {RoW}| market for sodium hydroxide, without	3.292×10^{-6}	kg	Lognormal	1.050	(1,2,5,5,1,na)

water, in 50% solution state ǀ Cut-off, U					
Solvent, organic {GLO}ǀ market for solvent, organic ǀ Cut-off, U	3.997×10^{-9}	kg	Lognormal	1.504	(1,1,5,1,1,na)
Steel, unalloyed {GLO}ǀ market for steel, unalloyed ǀ Cut-off, U	3.382×10^{-5}	kg	Lognormal	1.504	(1,1,5,1,1,na)
Tap water {RoW}ǀ market for tap water ǀ Cut-off, U	0.022	kg	Lognormal	1.050	(1,2,5,5,1,na)
Vanadium bearing magnetite (Magnetite 72% Fe, 2.2% V2O5)	1.530	kg	undefined		
Electricity, high voltage {GLO}ǀ market group for electricity, high voltage ǀ Cut-off, U	0.003	kWh	Lognormal	1.050	(1,2,5,5,1,na)
Electricity, medium voltage {GLO}ǀ market group for electricity, medium voltage ǀ Cut-off, U	0.025	kWh	Lognormal	1.506	(1,2,5,3,1,na)
Heat, district or industrial, natural gas {RoW}ǀ market for heat, district or industrial, natural gas ǀ Cut-off, U	0.066	MJ	Lognormal	1.506	(1,2,5,3,1,na)
Heat, district or industrial, other than natural gas {RoW}ǀ market for heat, district or industrial, other than natural gas ǀ Cut-off, U	0.023	MJ	Lognormal	1.504	(1,1,5,1,1,na)

Table S5. Vanadium Slag (25% V_2O_5) production unit process inventory.

Output	Amount	Unit	Distribution	SD	Comments
Vanadium Slag (25% V_2O_5)	0.061	kg	undefined		
Steel, low-alloyed	1.200	kg	undefined		
Benzene	2.307×10^{-6}	kg	undefined		
Cadmium (II)	3.65×10^{-8}	kg	undefined		
Carbon monoxide, fossil	0.002	kg	undefined		
Chromium, ion	1.254×10^{-6}	kg	undefined		
Copper, ion	2.305×10^{-7}	kg	undefined		
Dioxins (TEQ)	4.535×10^{-12}	kg	undefined		
Hydrocarbons, aromatic	7.701×10^{-5}	kg	undefined		
Hydrochloric acid	5.200×10^{-6}	kg	undefined		
Hydrogen fluoride	2.350×10^{-6}	kg	undefined		
Lead (II)	1.808×10^{-6}	kg	undefined		
Mercury (II)	2.224×10^{-6}	kg	undefined		
Nickel (II)	7.005×10^{-7}	kg	undefined		
Nitrogen oxides	1.800×10^{-4}	kg	undefined		
PAHs, polycyclic aromatic hydrocarbons	3.725×10^{-8}	kg	undefined		
Particulates, < 2.5 um	1.657×10^{-4}	kg	undefined		
Particulates, > 10 um	5.857×10^{-5}	kg	undefined		
Particulates, > 2.5 um, and < 10um	1.6596	kg	undefined		
Polybrominated biphenyls	2.325×10^{-8}	kg	undefined		
Sulfur dioxide	7.700×10^{-5}	kg	undefined		
Water	2.021×10^{-3}	kg	undefined		

Zinc (II)	2.294×10^{-5}	kg	undefined	
Argon-40/kg	0.003	kg	undefined	
Benzene, hexachloro-	2.000×10^{-8}	kg	undefined	
**Emissions to water**				
Water	03.19×10^{-3}	kg	undefined	
Chloride	1.800×10^{-6}	kg	undefined	
Chromium (VI)	1.900×10^{-9}	kg	undefined	
Inert waste, for final disposal {RoW}∣ market for inert waste, for final disposal ∣ Cut-off, U	0.005	kg	undefined	
Electric arc furnace dust {RoW}∣ market for electric arc furnace dust ∣ Cut-off, U	9.600×10^{-3}	kg	undefined	
Electric arc furnace slag {RoW}∣ market for electric arc furnace slag ∣ Cut-off, U	0.049	kg	undefined	
Scrap steel {RoW}∣ market for scrap steel ∣ Cut-off, U	5.200×10^{-5}	kg	undefined	
Spent solvent mixture {Europe without Switzerland}∣ market for spent solvent mixture ∣ Cut-off, U	3.300×10^{-8}	kg	undefined	
1 kg blast furnace slag transport	0.093	p	undefined	
**Input**				
Water, cooling, unspecified natural origin, RoW	5.216×10^{-3}	m^3	undefined	
Anode, for metal electrolysis {GLO}∣ market for anode, for metal electrolysis ∣ Cut-off, U	0.003	kg	undefined	
Electric arc furnace converter {GLO}∣ market for electric arc furnace converter ∣ Cut-off, U	4.000×10^{-11}	p	undefined	
Hard coal {CN}∣ market for hard coal ∣ Cut-off, U	0.014	kg	undefined	
Natural gas, high pressure {RoW}∣ market for natural gas, high pressure ∣ Cut-off, U	0.025	m3	undefined	
Oxygen, liquid {RoW}∣ market for oxygen, liquid ∣ Cut-off, U	0.051	kg	undefined	
Quicklime, in pieces, loose {RoW}∣ market for quicklime, in pieces, loose ∣ Cut-off, U	0.055	kg	undefined	
Refractory, basic, packed {GLO}∣ market for refractory, basic, packed ∣ Cut-off, U	0.014	kg	undefined	
Pre-reduced V2O5 magnetite production ∣ Cut-off, U	1.460	kg	undefined	
Aluminium, wrought alloy {GLO}∣ market for aluminium, wrought alloy ∣ Cut-off, U	1.500×10^{-5}	kg	undefined	
Argon, liquid {RoW}∣ market for argon, liquid ∣ Cut-off, U	0.003	kg	undefined	

Cast iron {GLO}∣ market for cast iron ∣ Cut-off, U	5.200 × 10^{-5}	kg	undefined
Diesel, burned in building machine {GLO}∣ market for diesel, burned in building machine ∣ Cut-off, U	0.003	MJ	undefined
Ethylene glycol {RoW}∣ market for ethylene glycol ∣ Cut-off, U	3.300 × 10^{-8}	kg	undefined
Ferrochromium, high-carbon, 68% Cr {GLO}∣ market for ferrochromium, high-carbon, 68% Cr ∣ Cut-off, U	1.100 × 10^{-4}	kg	undefined
Ferromanganese, high-coal, 74.5% Mn {GLO}∣ market for ferromanganese, high-coal, 74.5% Mn ∣ Cut-off, U	4.500 × 10^{-5}	kg	undefined
Ferrosilicon {GLO}∣ market for ferrosilicon ∣ Cut-off, U	0.004	kg	undefined
Molybdenum trioxide {GLO}∣ market for molybdenum trioxide ∣ Cut-off, U	1.400 × 10^{-5}	kg	undefined
Nickel, class 1 {GLO}∣ market for nickel, class 1 ∣ Cut-off, U	3.300 × 10^{-5}	kg	undefined
Propane, burned in building machine {GLO}∣ market for propane, burned in building machine ∣ Cut-off, U	0.003	MJ	undefined
Electricity, medium voltage {CN}∣ market group for electricity, medium voltage ∣ Cut-off, U	0.540	kWh	undefined
Electricity, low voltage {CN}∣ market group for electricity, low voltage ∣ Cut-off, U	0.034	kWh	undefined
Heat, district or industrial, natural gas {RoW}∣ market for heat, district or industrial, natural gas ∣ Cut-off, U	1.230	MJ	undefined

Table S6. Vanadium Pentaoxide (V_2O_5) production unit process inventory.

Output	Amount	Unit	Distribution	SD	Comments
Vanadium Pentaoxide V_2O_5	1.000	kg			
Sodium sulfate, anhydrite	1.000	kg	undefined		
Carbon dioxide	0.160	kg	lognormal	1.513	(2,3,5,3,1,na)
Oxygen	0.056	kg	undefined		
Sulfur dioxide	0.230	kg	lognormal	5.015	(2,3,5,3,1,na)
Water	0.085	kg	lognormal	5.015	(2,2,5,1,1,na)
**Input**					
Vanadium Slag (25% V_2O_5)	1.350	kg	undefined		
Ammonium sulfate {RER}∣ market for ammonium sulfate ∣ Cut-off, U	0.310	kg	undefined		
Soda ash, dense {GLO}∣ market for soda ash, dense ∣ Cut-off, U	0.370	kg	undefined		
Sodium sulfate, anhydrite {RoW}∣ market for sodium sulfate, anhydrite ∣ Cut-off, U	0.500	kg	undefined		

Sulfuric acid {RoW}∣ market for sulfuric acid ∣ Cut-off, U	0.460	kg	lognormal	1.491	(3,2,5,5,1,na)
Transport, freight train {CN}∣ market for transport, freight train ∣ Cut-off, U	0.460	tkm	lognormal		
Transport, freight, lorry 16-32 metric ton, EURO6 {RER}∣ transport, freight, lorry 16-32 metric ton, EURO6 ∣ Cut-off, U	0.077	tkm	undefined		
Water, deionised {RoW}∣ market for water, deionised ∣ Cut-off, U	3.160	kg	undefined		
Electricity, medium voltage {CN}∣ market group for electricity, medium voltage ∣ Cut-off, U	0.200	kWh	lognormal	1.155	(3,3,1,5,1,na)
Heat, district or industrial, natural gas {RoW}∣ market for heat, district or industrial, natural gas ∣ Cut-off, U	0.930	MJ	lognormal	1.505	(1,2,5,3,1,na)

Table S7. Vanadium chloride (VCl_3) production unit process inventory [3].

**Output**	**Amount**	**Unit**	**Distribution**	**SD**	**Comments**
Vanadium Chloride VCl_3	1.000	kg			
Water	0.263	kg			
**Input**					
Vanadium Pentaoxide V_2O_5	0.578	kg	lognormal		
Hydrochloric acid, without water, in 30% solution state {RoW}∣ market for hydrochloric acid, without water, in 30% solution state ∣ Cut-off, U	0.695	kg	lognormal	1.492	(3,2,5,5,1,na)
Hydrogen, gaseous, medium pressure, merchant {RoW}∣ market for hydrogen, gaseous, medium pressure, merchant ∣ Cut-off, U	0.006	kg	undefined		
Electricity, medium voltage {CN}∣ market group for electricity, medium voltage ∣ Cut-off, U	0.330	kWh	lognormal	1.155	(3,3,1,5,1,na)

Table S8. Vanadium unrefined production unit process inventory. This process was adapted from "Titanium sponge {RoW}∣ titanium sponge production, from titanium tetrachloride ∣ Cut-off, U" [2].

**Output**	**Amount**	**Unit**	**Distribution**	**SD**	**Comments**
Vanadium unrefined production, from vanadium trichloride	4.00×10^{-10}	p	Lognormal	3.059	(3,3,1,5,3,na)
**Input**					
Chemical factory, organics {GLO}∣ market for chemical factory, organics ∣ Cut-off, U	4.000×10^{-10}	p	Lognormal	3.059	(3,3,1,5,3,na)
Magnesium {GLO}∣ market for magnesium ∣ Cut-off, U	28.590	g	Lognormal	1.155	(3,3,1,5,1,na)

Magnesium chloride, from titanium sponge production {GLO}∣ magnesium chloride, from titanium sponge production, Recycled Content cut-off ∣ Cut-off, U	−2737.710	g	Lognormal	1.155	(3,3,1,5,1,na)
Magnesium, for reuse in titanium sponge production {RoW}∣ market for magnesium, for reuse in titanium sponge production ∣ Cut-off, U	687.030	g	Lognormal	1.155	(3,3,1,5,1,na)
Vanadium Chloride VCl3	2391.710	g	Lognormal	1.155	(3,3,1,5,1,na)
Electricity, medium voltage {AM}∣ market for electricity, medium voltage ∣ Cut-off, U	1.950	Wh	Lognormal	1.155	(3,3,1,5,1,na)
Electricity, medium voltage {AU}∣ market for electricity, medium voltage ∣ Cut-off, U	66.580	Wh	Lognormal	1.155	(3,3,1,5,1,na)
Electricity, medium voltage {AZ}∣ market for electricity, medium voltage ∣ Cut-off, U	6.480	Wh	Lognormal	1.155	(3,3,1,5,1,na)
Electricity, medium voltage {BD}∣ market for electricity, medium voltage ∣ Cut-off, U	21.280	Wh	Lognormal	1.155	(3,3,1,5,1,na)
Electricity, medium voltage {BH}∣ market for electricity, medium voltage ∣ Cut-off, U	5.480	Wh	Lognormal	1.155	(3,3,1,5,1,na)
Electricity, medium voltage {BN}∣ market for electricity, medium voltage ∣ Cut-off, U	1.150	Wh	Lognormal	1.155	(3,3,1,5,1,na)
Electricity, medium voltage {CY}∣ market for electricity, medium voltage ∣ Cut-off, U	1.470	Wh	Lognormal	1.155	(3,3,1,5,1,na)
Electricity, medium voltage {GE}∣ market for electricity, medium voltage ∣ Cut-off, U	3.490	Wh	Lognormal	1.155	(3,3,1,5,1,na)
Electricity, medium voltage {HK}∣ market for electricity, medium voltage ∣ Cut-off, U	14.790	Wh	Lognormal	1.155	(3,3,1,5,1,na)
Electricity, medium voltage {ID}∣ market for electricity, medium voltage ∣ Cut-off, U	54.530	Wh	Lognormal	1.155	(3,3,1,5,1,na)
Electricity, medium voltage {IL}∣ market for electricity, medium voltage ∣ Cut-off, U	17.810	Wh	Lognormal	1.155	(3,3,1,5,1,na)
Electricity, medium voltage {IN}∣ market group for electricity, medium voltage ∣ Cut-off, U	403.220	Wh	Lognormal	1.155	(3,3,1,5,1,na)
Electricity, medium voltage {JO}∣ market for electricity, medium voltage ∣ Cut-off, U	4.990	Wh	Lognormal	1.155	(3,3,1,5,1,na)
Electricity, medium voltage {KG}∣ market for electricity, medium voltage ∣ Cut-off, U	4.400	Wh	Lognormal	1.155	(3,3,1,5,1,na)

Table S9. Vanadium unrefined market unit process inventory. This is the market for vanadium unrefined production and was adapted from "Titanium sponge {GLO}| market for titanium sponge | Cut-off, U".

Output	Amount	Unit	Distribution	SD	Comments
Vanadium unrefined	1.000	kg			Properties<br>Carbon content, fossil: 0 kg C/kg dry mass<br>Carbon content, non-fossil: 0 kg C/kg dry mass<br>Dry mass: 1 kg<br>Wet mass: 1 kg
**Input**					
Vanadium unrefined production, from vanadium trichloride	1.000	kg	Lognormal	3.059	(3,3,1,5,3,na)
Transport, freight train {GLO}| market group for transport, freight train | Cut-off, U	0.345	tkm	Lognormal	2.248	(1,1,3,5,4,na)
Transport, freight, inland waterways, barge {GLO}| market group for transport, freight, inland waterways, barge | Cut-off, U	0.036	tkm	Lognormal	2.248	(1,1,3,5,4,na)
Transport, freight, lorry, unspecified {GLO}| market group for transport, freight, lorry, unspecified | Cut-off, U	0.361	tkm	Lognormal	2.248	(1,1,3,5,4,na)

Table S10. Vanadium production unit process inventory. This process was adapted from titanium triple-melt production and represents the melting purification process [1].

Output	Amount	Unit	Distribution	SD	Comments
Vanadium, triple-melt production	1.000	kg			
**Input**					
Vanadium unrefined market for | Cut-off, U	1.000	kg	Lognormal	2.504	(5,5,5,5,5,na)<br>**This matrix was manually inserted.**
Electricity, high voltage {GLO}| market group for electricity, high voltage | Cut-off, U	4.000	kW	Lognormal	1.492	(1,1,5,1,1,na)

Table S11. Vanadium market unit process inventory. This is the market for "Vanadium, triple-melt production" was adapted from "Titanium, triple-melt {GLO}| market for titanium, triple-melt".

Output	Amount	Unit	Distribution	SD	Comments
Vanadium triple-melt	1.000	kg			

**Input**					
Vanadium, triple-melt production	1.000	kg	Lognormal	1.890	(4,5,5,5,4,na) **This matrix was manually inserted.**
Transport, freight train {GLO}丨 market group for transport, freight train 丨 Cut-off, U	0.345	tkm	Lognormal	2.248	(1,1,3,5,4,na)
Transport, freight, inland waterways, barge {GLO}丨 market group for transport, freight, inland waterways, barge 丨 Cut-off, U	0.036	tkm	Lognormal	2.248	(1,1,3,5,4,na)
Transport, freight, lorry, unspecified {GLO}丨 market group for transport, freight, lorry, unspecified 丨 Cut-off, U	0.361	tkm	Lognormal	2.248	(1,1,3,5,4,na)
Transport, freight, sea, container ship {GLO}丨 market for transport, freight, sea, container ship 丨 Cut-off, U	0.363	tkm	Lognormal	2.248	(1,1,3,5,4,na)

Table S12. LCA characterization results (EF 3.1 method) and uncertainty (100 simulations) with the FU of 1 kg of each refined metal present in the membrane compositions (95% confidence interval).

**Impact Category**	**Unit**	**Pd**		**Ti**		**V**		**Zr**		**Cr**		**Ag**	
		Mean	SD	Mean	SD	Mean	SD	Mean	SD	Mean	SD	Mean	SD
Acidification	mol H^+ eq	2.21×10^{4}	4.84×10^{2}	3.23×10^{-1}	1.64×10^{-1}	6.58×10^{-1}	3.87×10^{-1}	2.07×10^{-1}	8.08×10^{-2}	1.35×10^{-1}	1.70×10^{-2}	5.38	1.11
Climate change	kg CO2 eq	1.12×10^{4}	1.79×10^{3}	5.19×10^{1}	2.50×10^{1}	4.67×10^{1}	2.64×10^{1}	3.34×10^{1}	1.28×10^{1}	2.73×10^{1}	2.94	4.75×10^{2}	5.10×10^{1}
Ecotoxicity, freshwater	CTUe	2.24×10^{5}	8.81×10^{4}	-1.85×10^{2}	4.75×10^{3}	3.94×10^{2}	4.53×10^{2}	3.37×10^{2}	2.02×10^{3}	1.11×10^{2}	1.48×10^{2}	3.59×10^{4}	9.34×10^{3}
Eutrophication, freshwater	kg P eq	1.37×10^{1}	6.49	2.41×10^{-2}	1.52×10^{-2}	1.38×10^{-2}	1.03×10^{-2}	1.96×10^{-2}	1.23×10^{-2}	1.03×10^{-2}	7.66×10^{-3}	1.30	3.76×10^{-1}
Eutrophication, marine	kg N eq	5.60×10^{1}	1.07×10^{1}	5.36×10^{-2}	2.45×10^{-2}	5.84×10^{-2}	3.28×10^{-2}	3.63×10^{-2}	1.46×10^{-2}	2.59×10^{-2}	3.15×10^{-3}	1.58	5.33×10^{-1}
Eutrophication, terrestrial	mol N eq	8.25×10^{2}	1.53×10^{2}	5.58×10^{-1}	2.58×10^{-1}	5.52×10^{-1}	3.12×10^{-1}	3.71×10^{-1}	1.48×10^{-1}	2.74×10^{-1}	3.28×10^{-2}	1.96×10^{1}	6.08
Human toxicity, cancer	CTUh	2.70×10^{4}	2.45×10^{-5}	8.35×10^{-8}	1.54×10^{-6}	3.14×10^{-1}	2.64×10^{-7}	1.90×10^{-7}	6.07×10^{-7}	1.69×10^{-7}	7.16×10^{-8}	1.24×10^{-6}	8.01×10^{-7}
Human toxicity, non-cancer	CTUh	2.44×10^{-4}	4.94×10^{-3}	-2.70×10^{-5}	3.56×10^{-4}	1.52×10^{-5}	2.97×10^{-5}	1.78×10^{-5}	1.48×10^{-4}	-8.23×10^{-7}	1.14×10^{-5}	1.53×10^{-5}	1.46×10^{-4}
Ionising radiation	kBq U-235 eq	6.53×10^{5}	5.96×10^{2}	3.96×10^{2}	5.21×10^{2}	3.97×10^{4}	4.15	6.87×10^{1}	3.92×10^{1}	2.80	2.82	5.03×10^{1}	5.18×10^{1}
Land use	Pt	9.27×10^{4}	4.89×10^{4}	2.44×10^{2}	1.17×10^{2}	1.20×10^{2}	6.68×10^{1}	2.93×10^{2}	1.30×10^{2}	5.93×10^{2}	1.24×10^{2}	5.56×10^{3}	3.45×10^{3}
Ozone depletion	kg CFC11 eq	8.58×10^{-5}	1.27×10^{-5}	1.09×10^{-6}	5.49×10^{-7}	5.53×10^{-7}	3.15×10^{-7}	8.06×10^{-7}	3.73×10^{-7}	2.76×10^{-7}	3.41×10^{-8}	5.06×10^{-6}	6.43×10^{-7}

Particulate matter	disease inc.	2.95×10^{-3}	4.88×10^{-4}	5.13×10^{-6}	2.81×10^{-6}	2.69×10^{-3}	1.62×10^{-3}	2.86×10^{-6}	1.40×10^{-6}	1.49×10^{-6}	7.93×10^{-7}	3.63×10^{-5}	9.56×10^{-6}
Photochemical ozone formation	kg NMVOC eq	2.98×10^{2}	4.35×10^{1}	2.01×10^{-1}	8.99×10^{-2}	1.99×10^{-1}	1.13×10^{-1}	2.07×10^{-1}	8.52×10^{-2}	8.59×10^{-2}	9.85×10^{-3}	4.66	1.40
Resource use, fossils	MJ	1.72×10^{5}	2.56×10^{4}	5.72×10^{2}	3.03×10^{2}	6.54×10^{2}	4.08×10^{2}	4.59×10^{2}	1.83×10^{2}	3.38×10^{2}	4.93×10^{1}	6.21×10^{3}	8.63×10^{2}
Resource use, minerals and metals	kg Sb eq	7.46×10^{-1}	6.91×10^{-2}	1.60×10^{-4}	1.09×10^{-4}	1.95×10^{-4}	1.18×10^{-4}	1.27×10^{-4}	8.09×10^{-5}	5.86×10^{-4}	1.35×10^{-4}	7.86×10^{-1}	1.13×10^{-1}
Water use	m3 depriv.	5.86×10^{3}	8.04×10^{4}	1.43×10^{2}	9.79×10^{2}	−4.66	1.85×10^{3}	-2.58×10^{1}	6.68×10^{2}	-4.69×10^{2}	1.94×10^{4}	1.57×10^{2}	1.14×10^{4}

Table S13. List of main environmental impact categories, their units of measurement and descriptions, gathered from the EN15804 standard and the Ecoinvent 3.10 database.

Impact Category	Unit	Description
Acidification	Kg mol H eq*	Indicator of the potential acidification of soils and water due to the release of gases such as nitrogen oxides and sulphur oxides
Climate Change	kg CO_2 eq	Indicator of potential global warming due to emissions of greenhouse gases to the air. Divided into 3 subcategories based on the emission source: (1) fossil resources, (2) bio-based resources and (3) land use change.
Ecotoxicity, freshwater (Ecotox., freshwater)	CTUe	Impact on freshwater organisms of toxic substances emitted to the environment.
Particulate matter	disease incidence	Indicator of the potential incidence of disease due to particulate matter emissions
Eutrophication, marine (Eutroph., marine)	kg N eq	Indicator of the enrichment of the marine ecosystem with nutritional elements, due to the emission of nitrogen-containing compounds.
Eutrophication, freshwater (Eutroph., freshwater)	kg PO_4 eq	indicator of the enrichment of the freshwater ecosystem with nutritional elements, due to the emission of nitrogen or phosphor-containing compounds
Eutrophication, terrestrial (Eutroph., terrestrial)	mol N eq	Indicator of the enrichment of the terrestrial ecosystem with nutritional elements, due to the emission of nitrogen-containing compounds.
Human toxicity, cancer and non-cancer (Hum. tox., canc. Hum. tox., non-canc.)	CTUh	Impact on humans of toxic substances emitted to the environment. Divided into non-cancer and cancer-related toxic substances.
Ionising radiation	kBq U-235 eq	Damage to human health and ecosystems linked to the emissions of radionuclides.
Land use	Pt	Measure of the changes in soil quality (Biotic production, Erosion resistance, Mechanical filtration).
Ozone depletion	kg CFC11 eq	Indicator of emissions to air that causes the destruction of the stratospheric ozone layer

Photochemical ozone formation (Photochem. oz. form.)	kg NMVOC eq	Indicators of emissions of gases that affect the creation of photochemical ozone in the lower atmosphere (smog) catalysed by sunlight.
Resource use, fossils (Res. Use, fossils)	MJ	Indicator of the depletion of natural fossil fuel resources.
Resource use, minerals and metals (Res. use, min. met.)	kg Sb eq	Indicator of the depletion of natural non-fossil resources.
Water use	m^3 deprived	Indicator of the relative amount of water used, based on regionalized water scarcity factors.

*eq: equivalent

Table S14. LCA inventory of the studied membranes. FU is thickness of 3.16 μm (equal to the PdAg one), and the LCA reference flow was the mass of each metallic membrane deposited with that thickness onto 2.56 cm² alumina substrate.

Membrane	Composition	Amount (kg)	Distribution	Mass Uncertainty (squared GSD)
PdAg	$Pd_{77}Ag_{23}$	9.67×10^{-6}	Lognormal	1.124
TiVCr	$Ti_{20}V_{33}Cr_{47}$	5.06×10^{-6}	Lognormal	1.173
	Pd coatings	1.24×10^{-6}	Lognormal	1.469
VPd	$V_{93}Pd_7$	7.16×10^{-6}	Lognormal	1.135
	Pd coatings	1.24×10^{-6}	Lognormal	1.469
ZrVTiPd1	$Zr_9V_{34}Ti_{30}Pd_{27}$	7.19×10^{-6}	Lognormal	1.079
	Pd coatings	1.24×10^{-6}	Lognormal	1.469
ZrVTiPd2	$Zr_{52}V_{12}Ti_{13}Pd_{23}$	7.59×10^{-6}	Lognormal	1.078
	Pd coatings	1.24×10^{-6}	Lognormal	1.469
ZrVTiPd3	$Zr_{39}V_{20}Ti_{19}Pd_{22}$	6.14×10^{-6}	Lognormal	1.084
	Pd coatings	1.24×10^{-6}	Lognormal	1.469

Table S15. LCA characterized results of the studied membranes, based on the $Pd_{77}Ag_{23}$ thickness (EF 3.1 method, and 95% confidence interval). The FU is thickness of 3.16 μm (equal to the PdAg one), and the LCA reference flow was the mass of each metallic membrane deposited with that thickness onto 2.56 cm² alumina substrate.

Impact Category	Unit	PdAg		VPd		TiVCr		TiZrVPd1		TiZrVPd2		TiZrVPd3	
		Mean	SD	Mean	SD	Mean	SD	Mean	SD	Mean	SD	Mean	SD
Acidification	mol H^+ eq	1.69×10^{-2}	4.04×10^{-3}	8.12×10^{-3}	2.12×10^{-3}	2.82×10^{-3}	8.97×10^{-4}	1.44×10^{-2}	3.37×10^{-3}	1.11×10^{-2}	2.92×10^{-3}	9.90×10^{-3}	2.51×10^{-3}
Climate change	kg CO2 eq	8.70×10^{-2}	1.47×10^{-2}	4.19×10^{-2}	7.30×10^{-3}	1.43×10^{-2}	3.43×10^{-3}	7.31×10^{-2}	1.19×10^{-2}	5.58×10^{-2}	9.30×10^{-3}	4.92×10^{-2}	8.33×10^{-3}
Ecotoxicity, freshwater	CTUe	1.76	7.00×10^{-1}	7.94×10^{-1}	3.37×10^{-1}	2.72×10^{-1}	1.32×10^{-1}	1.39	6.16×10^{-1}	1.05	4.77×10^{-1}	9.35×10^{-1}	4.08×10^{-1}
Eutrophication, freshwater	kg P eq	9.94×10^{-5}	4.04×10^{-5}	4.71×10^{-5}	2.01×10^{-5}	1.64×10^{-5}	8.11×10^{-6}	8.33×10^{-5}	3.61×10^{-5}	6.41×10^{-5}	2.74×10^{-5}	5.70×10^{-5}	2.84×10^{-5}
Eutrophication, marine	kg N eq	4.23×10^{-4}	8.89×10^{-5}	2.04×10^{-4}	4.45×10^{-5}	7.09×10^{-5}	2.12×10^{-5}	3.65×10^{-4}	7.71×10^{-5}	2.77×10^{-4}	5.95×10^{-5}	2.47×10^{-4}	5.42×10^{-5}

Eutrophication, terrestrial	mol N eq	6.16×10^{-3}	1.23×10^{-3}	2.98×10^{-3}	6.19×10^{-4}	1.03×10^{-3}	2.95×10^{-4}	5.32×10^{-3}	1.04×10^{-3}	4.03×10^{-3}	8.18×10^{-4}	3.60×10^{-3}	7.45×10^{-4}
Human toxicity, cancer	CTUh	1.97×10^{-10}	1.81×10^{-10}	9.09×10^{-11}	9.11×10^{-11}	3.15×10^{-11}	3.41×10^{-11}	1.68×10^{-10}	1.78×10^{-10}	1.21×10^{-10}	1.20×10^{-10}	1.10×10^{-10}	1.13×10^{-10}
Human toxicity, non-cancer	CTUh	2.64×10^{-9}	3.91×10^{-8}	-3.71×10^{-10}	1.95×10^{-8}	-5.78×10^{-11}	7.00×10^{-9}	1.33×10^{-9}	3.75×10^{-8}	-2.67×10^{-10}	2.63×10^{-8}	6.60×10^{-10}	2.41×10^{-8}
Ionising radiation	kBq U-235 eq	4.62×10^{-3}	4.20×10^{-3}	2.13×10^{-3}	1.86×10^{-3}	7.97×10^{-4}	8.32×10^{-4}	4.05×10^{-3}	3.70×10^{-3}	3.57×10^{-3}	3.67×10^{-3}	3.02×10^{-3}	3.31×10^{-3}
Land use	Pt	6.90×10^{-1}	3.92×10^{-1}	3.19×10^{-1}	1.81×10^{-1}	1.15×10^{-1}	6.83×10^{-2}	5.81×10^{-1}	3.40×10^{-1}	4.32×10^{-1}	2.41×10^{-1}	3.88×10^{-1}	2.15×10^{-1}
Ozone depletion	kg CFC11 eq	6.46×10^{-10}	1.01×10^{-10}	3.13×10^{-10}	4.97×10^{-11}	1.08×10^{-10}	2.52×10^{-11}	5.51×10^{-10}	7.83×10^{-11}	4.20×10^{-10}	6.31×10^{-11}	3.70×10^{-10}	5.61×10^{-11}
Particulate matter	disease inc.	2.22×10^{-8}	4.20×10^{-9}	5.12×10^{-8}	2.47×10^{-8}	1.20×10^{-8}	5.75×10^{-9}	2.75×10^{-8}	6.25×10^{-9}	1.68×10^{-8}	3.21×10^{-9}	1.66×10^{-8}	3.37×10^{-9}
Photochemical ozone formation	kg NMVOC eq	2.26×10^{-3}	3.61×10^{-4}	1.09×10^{-3}	1.95×10^{-4}	3.77×10^{-4}	9.70×10^{-5}	1.94×10^{-3}	3.09×10^{-4}	1.48×10^{-3}	2.51×10^{-4}	1.32×10^{-3}	2.26×10^{-4}
Resource use, fossils	MJ	1.27	1.94×10^{-1}	6.16×10^{-1}	9.79×10^{-2}	2.09×10^{-1}	4.88×10^{-02}	1.09	1.63×10^{-1}	8.19×10^{-1}	1.27×10^{-1}	7.24×10^{-1}	1.11×10^{-1}
Resource use, minerals and metals	kg Sb eq	7.39×10^{-6}	7.08×10^{-7}	2.72×10^{-6}	3.39×10^{-7}	9.20×10^{-7}	2.00×10^{-7}	4.83×10^{-6}	5.02×10^{-7}	3.63×10^{-6}	3.97×10^{-7}	3.24×10^{-6}	3.55×10^{-7}
Water use	m3 depriv.	8.59×10^{-3}	5.79×10^{-1}	-5.90×10^{-3}	2.76×10^{-1}	1.94×10^{-3}	1.51×10^{-1}	1.10×10^{-2}	4.65×10^{-1}	2.06×10^{-3}	3.54×10^{-1}	1.64×10^{-2}	3.18×10^{-1}

Table S16. Uncertainty analysis results for varying numbers of Monte Carlo simulations (95% confidence interval), comparison of the single score results of the studied membranes and their uncertainty range. The FU is thickness of 3.16 μm (equal to the PdAg one), and the LCA reference flow was the mass of each metallic membrane deposited with that thickness onto 2.56 cm^2 alumina substrate. The unit is μPt.

**Membrane/Number of runs**		**100**	**1000**	**10,000**
$Pd_{77}Ag_{23}$	Mean	4.32×10^{-5}	4.21×10^{-5}	4.22×10^{-5}
	SD	9.60 B0^{-6}	9.65×10^{-6}	1.05×10^{-5}
	Lower bound	1.83×10^{-5}	1.73×10^{-5}	1.81×10^{-5}
	Upper bound	1.87×10^{-5}	2.05×10^{-5}	2.05×10^{-5}
$V_{93}Pd_7$	Mean	1.45×10^{-5}	1.46×10^{-5}	1.46×10^{-5}
	SD	3.43×10^{-6}	5.99×10^{-6}	6.19×10^{-6}
	Lower bound	4.99×10^{-6}	5.88×10^{-6}	5.91×10^{-6}
	Upper bound	5.56×10^{-6}	7.89×10^{-6}	8.09×10^{-6}
$Ti_{20}V_{33}Cr_{47}$	Mean	7.25×10^{-6}	7.26×10^{-6}	7.24×10^{-6}
	SD	2.27×10^{-6}	2.39×10^{-6}	2.36×10^{-6}
	Lower bound	4.05×10^{-6}	3.62×10^{-6}	3.50×10^{-6}
	Upper bound	5.25×10^{-6}	5.04×10^{-6}	5.12×10^{-6}
$Ti_{30}Zr_9V_{34}Pd_{27}$	Mean	2.36×10^{-5}	2.37×10^{-5}	2.37×10^{-5}
	SD	6.0×10^{-6}	8.41×10^{-6}	8.33×10^{-6}

	Lower bound	1.15×10^{-5}	9.26×10^{-6}	9.99×10^{-6}
	Upper bound	9.48×10^{-6}	1.17×10^{-5}	1.19×10^{-5}
$Ti_{30}Zr_9V_{34}Pd_{23}$	Mean	1.91×10^{-5}	1.85×10^{-5}	1.88×10^{-5}
	SD	4.88×10^{-6}	6.30×10^{-6}	6.17×10^{-6}
	Lower bound	8.16×10^{-6}	7.77×10^{-6}	7.95×10^{-6}
	Upper bound	6.70×10^{-6}	9.58×10^{-6}	9.69×10^{-6}
$Ti_{30}Zr_9V_{34}Pd_{22}$	Mean	1.66×10^{-5}	1.70×10^{-5}	1.70×10^{-5}
	SD	3.96×10^{-6}	5.74×10^{-6}	5.64×10^{-6}
	Lower bound	7.61×10^{-6}	7.26×10^{-6}	7.37×10^{-6}
	Upper bound	9.61×10^{-6}	8.3×10^{-6}	8.95×10^{-6}

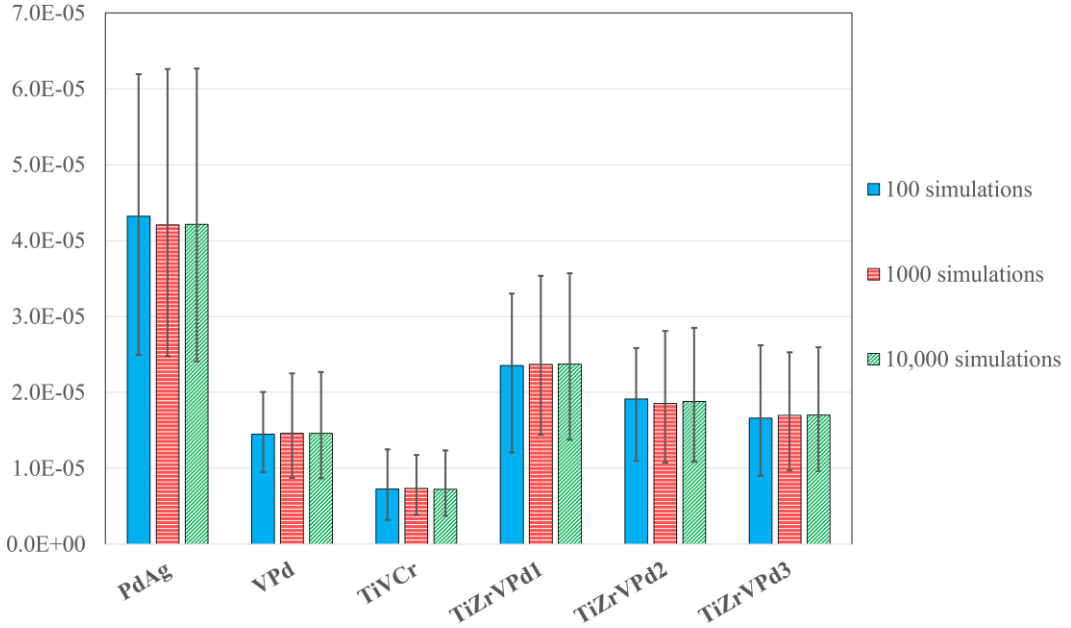

Figure S1. Comparison of the single score results (EF 3.1 method) and the uncertainty range of the studied membranes after different numbers of Monte Carlo calculations. FU: thickness of 3.16 μm (equal to the PdAg one) and the LCA reference flow was the mass of each metallic membrane deposited with that thickness onto 2.56 cm² alumina substrate).

Table S17. LCA characterization results of comparing the VPd and PdAg membranes The functional unit is the PdAg membrane permeance (2.03×10^{-6} mol m^{-2} Pa^{-1} s^{-1} for a 3.16 μm thick membrane), and the LCA reference flow of PdV was the mass of the membrane deposited with a thickness of 2.66 μm onto 2.56 cm² alumina substrate.

**Impact Category**	**Unit**	**PdAg**		**VPd**	
		**Mean**	**SD**	**Mean**	**SD**
Acidification	mol H^+ eq	1.69×10^{-2}	4.04×10^{-3}	4.44×10^{-3}	1.25×10^{-3}
Climate change	kg CO2 eq	8.70×10^{-2}	1.47×10^{-2}	2.23×10^{-2}	4.49×10^{-3}
Ecotoxicity, freshwater	CTUe	1.76	7.00×10^{-1}	4.07×10^{-1}	1.93×10^{-1}
Eutrophication, freshwater	kg P eq	9.94×10^{-5}	4.04×10^{-5}	2.59×10^{-5}	1.00×10^{-5}
Eutrophication, marine	kg N eq	4.23×10^{-4}	8.89×10^{-5}	1.05×10^{-4}	2.26×10^{-5}
Eutrophication, terrestrial	mol N eq	6.16×10^{-3}	1.23×10^{-3}	1.54×10^{-3}	3.07×10^{-4}

Human toxicity, cancer	CTUh	1.97×10^{-10}	1.81×10^{-10}	5.33×10^{-11}	4.49×10^{-11}
Human toxicity, non-cancer	CTUh	2.64×10^{-9}	3.91×10^{-8}	2.98×10^{-10}	9.39×10^{-9}
Ionising radiation	kBq U-235 eq	4.62×10^{-3}	4.20×10^{-3}	1.19×10^{-3}	1.75×10^{-3}
Land use	Pt	6.90×10^{-1}	3.92×10^{-1}	1.51×10^{-1}	9.46×10^{-2}
Ozone depletion	kg CFC11 eq	6.46×10^{-10}	1.01×10^{-10}	1.70×10^{-10}	3.28×10^{-11}
Particulate matter	disease inc.	2.22×10^{-8}	4.20×10^{-9}	1.71×10^{-8}	7.63×10^{-9}
Photochemical ozone formation	kg NMVOC eq	2.26×10^{-3}	3.61×10^{-4}	5.76×10^{-4}	1.12×10^{-4}
Resource use, fossils	MJ	1.27	1.94×10^{-1}	3.27×10^{-1}	5.75×10^{-2}
Resource use, minerals and metals	kg Sb eq	7.39×10^{-6}	7.08×10^{-7}	1.46×10^{-6}	2.07×10^{-7}
Water use	m^3 depriv.	8.59×10^{-3}	5.79×10^{-1}	-2.86×10^{-3}	1.46×10^{-1}

References

1. Norgate, T., Rajakumar, V., & Trang, S. (2004). Titanium and other light metals - Technology pathways to sustainable development. 105–112.
2. Gao, F.; Nie, Z.; Yang, D.; Sun, B.; Liu, Y.; Gong, X.; Wang, Z. Environmental impacts analysis of titanium sponge production using Kroll process in China. *J. Clean. Prod.* **2018**, *174*, 771–779, https://doi.org/10.1016/j.jclepro.2017.09.240.
3. Da Silva Lima, L.; Quartier, M.; Buchmayr, A.; Sanjuan-Delmás, D.; Laget, H.; Corbisier, D.; Mertens, J.; Dewulf, J. Life cycle assessment of lithium-ion batteries and vanadium redox flow batteries-based renewable energy storage systems. *Sustain. Energy Technol. Assess.* **2021**, *46*, 101286, https://doi.org/10.1016/j.seta.2021.101286.
